# Untargeted metabolite profiling to elucidate rhizosphere and leaf metabolome changes of wheat cultivars (*Triticum aestivum* L.) treated with the plant growth-promoting rhizobacteria *Paenibacillus alvei* (T22) and *Bacillus subtilis*

**DOI:** 10.3389/fmicb.2022.971836

**Published:** 2022-08-25

**Authors:** Manamele D. Mashabela, Fidele Tugizimana, Paul A. Steenkamp, Lizelle A. Piater, Ian A. Dubery, Msizi I. Mhlongo

**Affiliations:** ^1^Research Centre for Plant Metabolomics, Department of Biochemistry, University of Johannesburg, Johannesburg, South Africa; ^2^International Research and Development Division, Omnia Group, Ltd., Johannesburg, South Africa

**Keywords:** *Bacillus subtilis*, exo-metabolome, metabolomics, *Paenibacillus alvei*, PGPR, rhizosphere

## Abstract

The rhizosphere is a highly complex and biochemically diverse environment that facilitates plant–microbe and microbe–microbe interactions, and this region is found between plant roots and the bulk soil. Several studies have reported plant root exudation and metabolite secretion by rhizosphere-inhabiting microbes, suggesting that these metabolites play a vital role in plant–microbe interactions. However, the biochemical constellation of the rhizosphere soil is yet to be fully elucidated and thus remains extremely elusive. In this regard, the effects of plant growth-promoting rhizobacteria (PGPR)–plant interactions on the rhizosphere chemistry and above ground tissues are not fully understood. The current study applies an untargeted metabolomics approach to profile the rhizosphere exo-metabolome of wheat cultivars generated from seed inoculated (bio-primed) with *Paenibacillus* (T22) and *Bacillus subtilis* strains and to elucidate the effects of PGPR treatment on the metabolism of above-ground tissues. Chemometrics and molecular networking tools were used to process, mine and interpret the acquired mass spectrometry (MS) data. Global metabolome profiling of the rhizosphere soil of PGPR-bio-primed plants revealed differential accumulation of compounds from several classes of metabolites including phenylpropanoids, organic acids, lipids, organoheterocyclic compounds, and benzenoids. Of these, some have been reported to function in plant–microbe interactions, chemotaxis, biocontrol, and plant growth promotion. Metabolic perturbations associated with the primary and secondary metabolism were observed from the profiled leaf tissue of PGPR-bio-primed plants, suggesting a distal metabolic reprograming induced by PGPR seed bio-priming. These observations gave insights into the hypothetical framework which suggests that PGPR seed bio-priming can induce metabolic changes in plants leading to induced systemic response for adaptation to biotic and abiotic stress. Thus, this study contributes knowledge to ongoing efforts to decipher the rhizosphere metabolome and mechanistic nature of biochemical plant–microbe interactions, which could lead to metabolome engineering strategies for improved plant growth, priming for defense and sustainable agriculture.

## Introduction

Plant roots exist in a composite ecosystem; in addition to functioning as anchors of plants into the soil, the roots serve as the central interface for plant–microbe interactions in the rhizosphere. Plants use root exudates as important mediators to facilitate reciprocal biochemical communications with the rhizosphere-inhabiting beneficial microorganisms (Pétriacq et al., 2017), leading to a delicately regulated microbial community. Furthermore, by changing the root microbiota at different developmental stages, the plant governs plant–soil–microbe feedback leading to changes in the rhizosphere chemistry over time which is crucial for survival under various environmental conditions (Korenblum et al., 2020). Over the years, scientists have applied sequencing technologies to isolate and characterize the rhizosphere microbial communities (Grobelak et al., 2015; Goswami et al., 2016). However, the dynamics and complexity of the rhizosphere plant–microbe interactions are largely unknown, and the chemical diversity of the rhizosphere remains essentially unexplored.

Plant growth-promoting rhizobacteria (PGPR) form the majority of free-living beneficial bacteria inhabiting the rhizosphere. The proximity of these microbes to the plant root system allows them to establish a mutual symbiotic relationship with the associated plant through biochemical communications (Grobelak et al., 2015; Mishra and Arora, 2017; Mhlongo et al., 2020; Mashabela et al., 2022a). The rhizosphere, as such, functions as the epicenter for biochemical signaling linking the PGPR and the associated plant roots. This region of the soil-root interface presents a favorable environment for PGPR accumulation and proliferation through a constant supply of nutritional and signaling compounds. The cocktail of the carbon-based compounds consists of organic acids, amino acids, vitamins and minerals, and allelochemicals, including phenolic acids, terpenoids and flavonoids produced and secreted by the plant as root exudates (Strehmel et al., 2014; Miller et al., 2019). In return, the PGPR produces and secretes plant-beneficial compounds (i.e., siderophoses, lipopeptides, and phytohormones) essential for defense signaling and plant growth promotion (Mus et al., 2016; Mhlongo et al., 2018). Mashabela et al. (2022a) highlights the modes of action plants use to recruit beneficial bacteria with the use of root exudates, where the types of exudates released by the plant may be dependent on the current nutritional or defense requirements of the plant and thus selectively populating the rhizosphere with the PGPR fit for utilization.

The rhizosphere chemistry undergoes continuous perturbations with the continuous flux of metabolites from the PGPR and the plant, which subsequently alters the metabolic pools of both organisms (Adeniji et al., 2020; Korenblum et al., 2020). In this respect, the organisms display favorable allelopathic properties (Li et al., 2020). PGPR produce and secrete primary growth promotion compounds such as carbohydrates, amino acids, proteins and signaling molecules, as well as secondary defense metabolites including volatile organic acids (VOCs) and phytohormones. These compounds effect a change in the metabolome of the plant, which leads to physiological modifications such as root elongation and cell wall stabilization (Noumavo et al., 2013; Lin et al., 2019). In addition, nutrient solubilization by PGPR further enhances nutrient uptake abilities of the plant, which lead to overall plant growth promotion through enhanced photosynthesis and biomass accumulation (Prasad et al., 2019).

Plant growth-promoting rhizobacteria-induced plant metabolic changes can further alter the host plant’s osmoregulation, oxidative stress management, biosynthetic pathways and the metabolism of phytohormones, leading to the transcriptional regulation of the plant’s stress response genes for plant fitness (Jochum et al., 2019; Korenblum et al., 2020). However, in studies focused on the characterization of rhizomicrobes and apparent effects on plant growth and protection, the complexity and dynamics of the rhizosphere metabolome and the resulting changes of rhizometabolites on plants are yet to be fully addressed. As such, the current study explores the diversity of the rhizosphere metabolome of PGPR-bio-primed wheat cultivars and the metabolic perturbations occurring in the plants due to PGPR inoculation compared to non-bio-primed plants. Here, ultra-high performance liquid chromatography-mass spectrometry (UHPLC-MS) was used to acquire raw data. MS data was further de-convoluted with applied multivariate data analysis tools (MVDA) to reveal the anticipated metabolite alterations and insights into the subsequent effects on the associated plant’s primary and secondary metabolic pathways.

## Materials and methods

**FIGURE 1:**
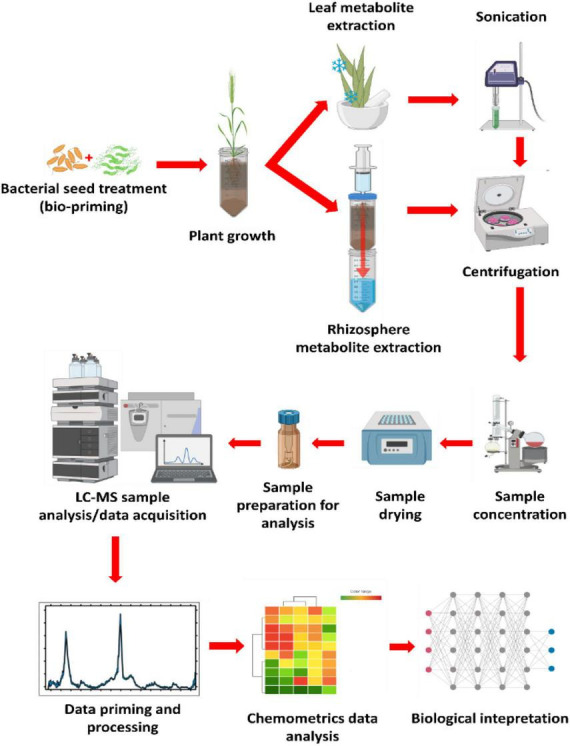
General experimental design and metabolomics workflow. The diagram above illustrates the basic experimental design utilized in the current study for the rhizosphere metabolite profiling after the seeds of the crop of interest were treated with strains of plant growth-promoting rhizobacteria (PGPR; bioprimed). The plants were grown in perforated conical centrifuge tubes. Metabolite extraction was carried out by pressurizing the extraction solvent (80% methanol) through the growth medium (rhizosphere) and collected in a clean centrifuge tube. Leaf metabolite extraction was done by crushing harvested leaves in liquid nitrogen (using a motar and a pestle), then sonicated with a probe sonicator from extraction. Both processes were followed by centrifugation to separate the metabolites from the rest of the extracted debris. Samples were then concentrated to 1 mL, then dried to completeness before reconstitution in 50% LC-grade methanol from sample analysis. This was followed by data mining, processing, chemometrics analysis, and biological interpretation.

In the last decade, various methods have been developed to collect and characterize root exudates from sterile and non-sterile rhizosphere soils. Here, a method applying untargeted metabolomics profiling of non-sterile rhizosphere exo-metabolome was adopted from Pétriacq et al. (2017) and adjusted to fit the objectives of this study. The experimental design consisted of four main elements as diagrammatically illustrated in Figure 1, namely (1) seed biopriming in which the seeds of wheat cultivars were treated with selected bacterial strains prior to planting; (2) plant cultivation, bioprimed seeds were planted in sand inside perforated centrifuge tubes for easier downstream metabolite extraction; (3) metabolite extraction using a modified lid attached to a syringe containing pressurized methanolic extraction solution, lastly (4) the methanolic extracts were analyzed on a UHPLC-ESI-Q-TOF-MS analytical instrument for data acquisition, followed by data mining, (pre-) processing and representation.

### Bacterial culture conditions

Two bacterial strains, *Paenibacillus alvei* (T22), obtained as glycerol stocks from Prof. N. Labuschagne, University of Pretoria, South Africa and *Bacillus subtilis* obtained from Omnia Group Ltd. (Bryanston, South Africa), were grown on Petri dishes with nutrient agar media overnight (O/N) at 28°C. *P. alvei* has been reported to induce a primed state in seedlings of *Sorghum bicolor* through induced systemic resistance (ISR) hence conferring protection against *Fusarium pseudograminearum* (Carlson et al., 2019). On the other hand, Qiao et al. (2017) reported the suppressive and biocontrol agent abilities of *B. subtilis* against *F. oxysprum*. Bacterial colonies of the two strains were transferred into a 50 mL Luria Broth (LB) culture medium for overnight incubation on a shaker at 140 rpm and 28°C. Overnight cultures optical density (OD) was adjusted to 0.5 OD_600_ for seed treatment. Bacterial cultures were centrifuged at 5000 rpm at 4°C for 15 min. The pellets were collected and gently washed in 5 mL sterile water, then reconstituted in 50 mL autoclaved water for seed treatment.

### Wheat seed biopriming and plant growth conditions

Two wheat cultivars of Gariep and Koonap were used in this study. The seeds were washed with autoclaved water and left to dry. Dried seeds were inoculated with previously prepared suspensions of the bacterial cultures; the seeds were immersed in separate 50 mL of reconstituted *P. alvei* and *B. subtilis* (OD_600_ 0.5) in centrifuge tubes and incubated for 3 h. Bacterial solutions were decanted, and seeds dried at 28°C for 24 h on open Petri dishes in an incubator. Control seeds were treated with sterile autoclaved water for the same period and dried under similar conditions. The seeds were then sown in washed sterile playpen sand in perforated growth (centrifuge) tubes approximately 1.4 cm below surface sand. Growth conditions were as follows; light/dark cycle of 12 h/12 h and light intensity of 60 μmol/m2/s at minimum temperature of 15°C and maximum temperature of 28°C. The plants were watered biweekly, once with distilled water and once with a fertilizer mixture consisting of 650 mg/L CaNO_3_, 400 mg/L KNO_3_, 300 mg/L MgSO_4_, 90 mg/L mono-ammonium phosphates, 90 mg/L mono-potassium phosphates, 150 mg/L Soluptase, 20 mg/L Microplex, and 40 μL/L Kep-P-Max obtained from Shiman SA (Olifantsfontein, South Africa). Each cultivar treatment was conducted in three independent biological replicates and samples were analyzed in triplicate (*n* = 9) to account for analytical variability (Mhlongo et al., 2020).

### Metabolite extraction

#### Rhizosphere metabolite extraction

Rhizosphere metabolite extraction was carried out from the first week post-germination for two consecutive weeks. An extraction method by Pétriacq et al. (2017) was adopted with minor modifications (Figure 1). A pressurized centrifuge tube lid was created by burning an approximately 4-mm hole on the lid, then fitted with a 60 ml syringe secured tight enough to allow maximum pressure to aid in running the extraction solvent through the growth tubes. A volume of 20 mL ice-cold 80% methanol was applied to the top of the growth tubes then flushed through the sand by applying pressure with the syringe. The analyte-containing extraction solution was collected in 50 mL clean centrifuge tubes followed by centrifugation at 5000 rpm for 15 min at 4°C to pellet the sand particles and recover extracts. The recovered supernatants were concentrated to approximately 1 mL at 55°C with a rotary evaporator. Concentrated samples were further dried at 55°C in a dry bath before reconstitution in 300 μL 50% LC grade methanol and filtered through a 0.22 μm nylon syringe into 2 mL LC vials fitted with a 500 μL insert and stored at 4°C until analysis.

#### Leaf metabolite extraction

Plant leaf extraction was done from week two post-germination for two consecutive weeks for a time-based metabolite profiling of the bioprimed plants to examine metabolite perturbations resulting from PGPR biopriming. Leaves were collected in 50 mL centrifuge tubes then flash-frozen in liquid nitrogen for metabolic quenching before extraction. A starting material of 1 g leaves were pulverized in liquid nitrogen, followed by resuspension in 10 mL 80% ice-cold methanol. The solution was sonicated twice with a probe sonicator at 55% power for 30 s each time at room temperature, followed by centrifugation at 5000 rpm for 15 min at 4°C. The supernatant was collected and evaporated to approximately 1 mL at 55°C with a rotary evaporator (Heidolph, Schwabach, DE). The extracts were dried to completeness in a vacuum microcentrifuge at 46°C, then reconstituted in 300 μL of 50% LC-grade methanol (Romil, Cambridge, United Kingdom) and filtered through 0.22 μm nylon syringe filters into 2 mL HPLC vials fitted with a 300 μL insert. Samples were stored at 4? until analysis. Quality control (QC) samples were prepared by pipetting equal volumes of the samples in a designated LC-MS vial for analysis.

### Metabolomics-based UHPLC-ESI-Q-TOF-MS data acquisition, analysis, and interpretation

Raw data acquisition was carried out as previously described by Mashabela et al. (2022b). A 2 μL volume per sample of the 50% methanol extracts was chromatographically separated on an HSS T3 column (1.7 μm × 2.2 mm × 150 mm; Waters, Manchester, United Kingdom), followed by detection of analytes on a SYNAPT G1 quadrupole time-of-flight high-definition mass spectrometer (Q-TOF-HD-MS, Waters, Manchester, United Kingdom) UHPLC system as follows: a binary mobile phase composed of water (eluent A) and acetonitrile (eluent B) (Romil Pure Chemistry, Cambridge, United Kingdom), both with 0.1% formic acid and 2.5% isopropyl alcohol (Sigma–Aldrich, Munich, Germany) were used at a flow rate of 0.4 mL/min for a run time of 30 min. The gradient was set as follows: eluent B ranged from 2% over the first 2.0 min, 2–90% over 2.0–25 min, 90–95% over 25–27 min, then returned from 95–2% over 28–30 min. Finally, the column was washed with a solution of methanol:acetonitrile:isopropyl alcohol (MeOH:ACN:IPA) for regeneration after batch analysis. Sample analysis was done in both positive and negative electron spray ionisation (ESI) source on a Waters SYNAPT G1 q-TOF MS with the following parameters: a 2.5 kV capillary voltage and 30 V sample cone voltage with an 1800 V MCP detector voltage, a 120°C source temperature, and a desolvation temperature of 450°C. The cone gas flow was set at 50 L/h, desolvation gas flow at 550 L/h, *m/z* range of 50–1200, a 0.1 s scan time in centroid mode with interscan delay: 0.02s, and a mass accuracy window of 0.5 Da. MassLynxTM 4.1 software (SCN 704, Waters Corporation Milford, MA, United States) was used to regulate the LC-MS run.

### Data mining and processing

Following data acquisition, the raw data was uploaded on MassLynx XM™ 4.1 software (Waters, Manchester, United Kingdom). The MarkerLynx™ multistep data pre-processing application manager allows for peak picking, retention time (Rt) correction and alignment, noise elimination, feature detection, and sample normalization, creating data matrices of Rt-*m/z* variable pairs, with *m/z* peak intensity for each sample (Mhlongo et al., 2020; Nephali et al., 2021; Mashabela et al., 2022b). The application manager further allows for data visualization, and pre-processing (including noise elimination and reduction of variables to acceptable parameters). The MarkerLynx™ parameters were restricted to process data within an Rt range of 0.68–26.35 min, a mass range of 100–1200 Da, with a noise elimination level of 10 applied for both ESI negative and positive data, the Rt difference and *m/z* tolerance of 0.2 min and 0.05 Da respectively were allowed for peak alignment observed across pooled samples. The total ion intensities were further used for data normalization. The processed matrices were exported for MetaboAnalyst compatible formatting in excel and uploaded to MetaboAnalyst 5.0^1^ for mining and remodeling to further explore the data structures and hidden underlying patterns. Data remodeling, using PCA and PLS-DA models, was carried out on MetaboAnalyst in conjunction with statistical analysis and model validation through evaluation of explained and predicted variation (cumulative *R*^2^ and *Q*^2^) (Nephali et al., 2021). Data deconvolution was further explored with statistical analysis and quantitative heatmaps to paint a clearer picture of the acquired data. Metabolite annotation was done on MS-based accurate mass and fragmentation patterns, existing compound spectral information generated from different collision energies of single ion extracted chromatograms (XICs) were consulted to derive molecular formulae of compounds of interest from full-scan accurate mass data. The selected molecular formulae were manually searched against bioinformatic databases such as Dictionary of Natural Products^2^, PubChem^3^, and ChemSpider^4^ taking into account the carefully inspected matching MS^1^ and MS*^E^* spectral data from putative metabolite annotation to a level 2 Metabolomics Standard Initiative (MSI) classification (Sumner et al., 2007; Mhlongo et al., 2016; Mareya et al., 2020). Selected metabolites were used for pathway analysis to paint a map of the metabolism subjected to the experimental conditions under investigation. Pathways analysis was done on MetPA (Metabolic Pathway Analysis) incorporated within the MetaboAnalyst tool kit, which uses previously established KEGG metabolic pathways as a source for pathway mapping.

### Molecular networking tools

The Reifys Abf converter software^5^ was used to convert raw vendor (Waters) MS/MS format files to analysis base file (ABF) file format compatible with Mass Spectrometry-Data Independent Analysis (MS-DIAL) software. Converted files were uploaded on the MS-DIAL software, a data-processing program which performs mass spectral deconvolution of data-independent acquisition (DIA) and data-dependent acquisition (DDA) centroid datasets for processing (Tsugawa et al., 2015; Othibeng et al., 2021). This software further combines main sources of information such as accurate mass, isotope ratios, Rt prediction, and MS/MS fragment matching to assist in compound identification and annotation (Tsugawa et al., 2015). Data processing was carried out as detailed by Othibeng et al. (2021) with minor modifications, i.e., a 0.05 Da mass accuracy (MS1 and MS2 tolerance), minimum peak height of 10 amplitude and mass slice width of 0.1 Da for peak detection, a sigma window value of 0.5 and a 0 MS/MS abundance cut-off for data deconvolution; a 0.20 min Rt tolerance was used under alignment parameter settings with one of the QC samples used as a reference file for alignment. Processed files were then exported as global natural product social (GNPS) export files (GnpsMgf and GnpsTable) were then uploaded into the GNPS environment^6^, along with metadata files using the WinSCP server for molecular networking. A metadata file describes the properties of the sample files (e.g., treatment and harvesting timepoint).

Using the respective workflow in the GNPS ecosystem, feature-based molecular network (FBMN) was computed for both positive and negative mode data. MN method clusters MS/MS (fragmentation) spectra using MS-cluster algorithms with a set precursor ion mass and fragment ion mass tolerance of 0.05 Da for a consensus spectra creation. For network generation, a minimum of 4 corresponding fragment ions and a cosine score of above 0.7 were specified for the edges connecting the nodes. A minimum of four corresponding fragment ions required for network generation allows for a more reliable collection of structurally related molecules giving rise to similar MS2 fragmentation patterns (Quinn et al., 2017; Aron et al., 2020). The generated molecular networks were then enhanced with the MolNetEnhancer workflow (Nothias et al., 2020) to improve structural annotations by integrating *in silico* tools such as substructure recognition topic modeling (MS2 latent Dirichlet allocation, MS2LDA, with MotifDB for annotated substructure patterns), Dereplicator+ and Network Annotation Propagation (NAP) for spectral annotation. MS2LDA decomposes molecular fragmentation data derived from large metabolomics experiments into annotated Mass2Motifs (van der Hooft et al., 2016), while Dereplicator+ is an algorithm for *in silico* identification of both peptidic and non-peptidic natural products (Othibeng et al., 2021). The computer networks were visualized in the Cytoscape network visualization software V3.8.2 (Othibeng et al., 2021).

Semi-automated metabolite annotation was carried out by searching the MS-DIAL metabolomics MSP spectral kits^7^ (public MS/MS libraries), using MS-DIAL software. Further automated metabolite annotation was performed by searching against GNPS libraries via FBMN, DEREPLICATOR and NAP searching mass spectral and structural databases such as GNPS, HMDB, FoodB, SUPNAT, CHEBI, and DRUGBANK. MS2LDA interface in GNPS was used to explore and annotate substructures. Manual metabolite annotation was further carried out to validate and improve the semi-automated annotations. This was carried out by inspecting fragmentation patterns (assessing the MS^1^ and MS*^E^* spectra) of the selected metabolite candidate and comparing against annotation information of metabolites reported in the literature.

## Results

### Untargeted metabolic profiles of rhizosphere soils and leaf extracts of plant growth-promoting rhizobacteria-bioprimed wheat cultivars by UHPLC-Q-TOF-HDMS

A global, untargeted metabolic profiling of rhizosphere soils of PGPR-bioprimed wheat cultivars was carried out. Rhizosphere samples were recovered with an extraction solution of 80% MeOH (methanol). An LC-MS analysis produced BPI chromatograms which provided visual and holistic representation of the complexity as well as differences and similarities in the metabolite profiles of the selected PGPR treatments and control samples. Supplementary Figures 1A,B show stacked chromatograms representing rhizosphere and leaf extractions from PGPR-treated (Gariep_*P. alvei* and Gariep_*B. sub*) and control (Gariep_untreated) samples respectively. Similar BPI chromatograms of PGPR-treated (Koonap_*P. alvei* and Koonap_*B. sub*) and control (Koonap_untreated) samples are shown in Supplementary Figures 2A,B respectively. The BPI chromatograms present clear differences in the qualitative (presence/absence) and quantitative (amount present) metabolic profiles of the samples. Additionally, leaf extractions resulted in higher quantities of detected metabolites in comparison to the rhizosphere, an indication of the varied diversity and complexity of metabolites associated with the endo- and exo-metabolome. Detected ion profiles were aligned and integrated using MassLynx XM™ 4.1 software (Waters Corporation) and further examined with multivariate data analysis using MetaboAnalyst 5.0^8^ to further extract biologically useful information through chemometric analysis.

### Multivariate data analysis of metabolite profiles from plant growth-promoting rhizobacteria-treated and untreated rhizosphere soils of wheat cultivars

**FIGURE 2:**
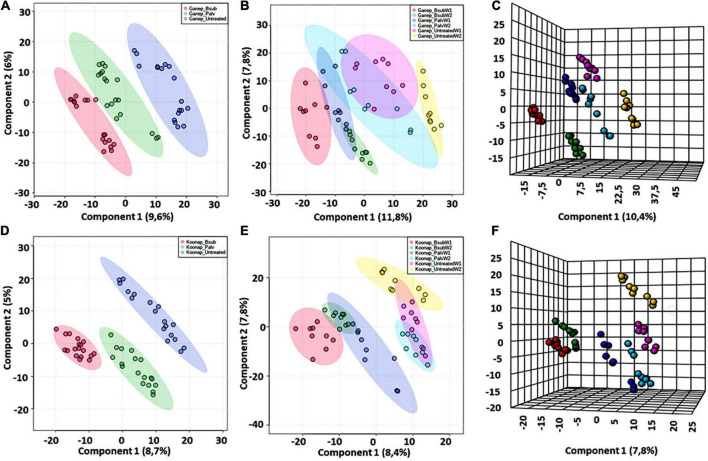
A supervised partial least square discriminant analysis (PLS-DA) models of plant growth-promoting rhizobacteria (PGPR)-treated and untreated wheat samples. **(A)** A PLS-DA scores plot showing the global differences in the metabolite profiles from rhizosphere extracts of PGPR-treated (red = Gariep_*B. sub*, green = Gariep_*P. alvei*) and untreated (blue) samples. **(B)** PLS-DA scores plot and corresponding 3D PLS-DA plot. **(C)** Shows the time-dependent variations and changes in the metabolite profiles of samples in **(A)**. GBW1 (red)/W2 (green), GPW1 (blue)/W2 (light blue), and GUW1 (purple)/W2 (yellow) represent the *B. sub-*treated, *P. alvei-*treated and untreated Gariep rhizosphere soils, respectively. **(D)** A PLS-DA scores plot showing the separation between plant growth-promoting rhizobacteria (PGPR)-treated (red = *B. sub*, green = *P. alvei*) and untreated (blue) rhizosphere samples of Koonap. The scores plots reveal the differential regulation of the rhizosphere exo-metabolome under varying bacterial treatments and the potential response mechanisms of the different cultivars. **(E,F)** Shows a 2D and 3D PLS-DA score plots of time-dependent variation in the metabolite profile of the Koonap cultivar from the first and second weeks of extractions respectively, where KBW1(red)/W2(green), KPW1(blue)/W2(light blue) and KUW1(purple)/W2(yellow) represent the *B. sub-*treated, *P. alvei-*treated and untreated Koonap rhizosphere soils, respectively. The data projected above were median-normalized, log transformed and *Pareto*-scaled in MetaboAnalyst for correlation and predictability scores of *R*2 = 0.946 and *Q*2 = 0.749 **(A)**; *R*2 = 0.916 and *Q*2 = 0.755 **(B)**, *R*2 = 0.984 and *Q*2 = 0.815 **(C)** and *R*2 = 0.952 and *Q*2 = 0.880; *R*2 = 0.928 and *Q*2 = 0.695 and *R*2 = 0.983 and *Q*2 = 0.754, for **(D–F)**, respectively.

Chemometric analysis of metabolomic data reduces the highly multi-dimensional and complex datasets, the applied pattern recognition and learning algorithms reveal trends, underlying structures and isolate significant features representing variations or similarities in the data matrix as described in (Pretorius et al., 2021). A supervised partial least squares discriminant analysis (PLS-DA) was used to compare metabolite profiles between PGPR-bioprimed (hence forth referred to as treated) and control rhizosphere extracts of both Koonap and Gariep cultivars (Figure 2). The corresponding PLS-DA models in displayed high correlation (*R*^2^) and predictability (*Q*^2^) scores.

The evaluation of the PLS-DA models in Figure 2 revealed clear treatment- and timepoint-based separations and grouping from the datasets. The underlying structures, groupings, and characteristics of the data were further correlated with the third components, showing clear separations from the 2D-PLS-DAs in Figures 2C,F. The separations observed give insight into the chemistry of the rhizosphere and the differential metabolite profiles occurring in response to interactions with varying types of microbes. This phenomenon can be indicated by the separation between control rhizosphere samples and rhizosphere samples treated with *P. alvei* (T22) and *B. sub* for both Gariep and Koonap cultivars. Figures 2A,D also show variations in the metabolite profiles in samples treated with *B. sub* compared to *P. alvei* (T22) treatment. Furthermore, the metabolite profiles in the rhizosphere showed to be differentially regulated over time as evidenced in Figures 2C,F, simply indicating that the data structures extracted by PLS-DA modeling point to underlying differences in the measured metabolite profiles in the different sample groups, which was then investigated further by applying tools for annotating metabolites and performing quantitative assessments below.

### Molecular networking tools to decipher the chemical space and the composition of the rhizosphere exo-metabolome from plant growth-promoting rhizobacteria-treated (seed bio-primed) wheat cultivars

**FIGURE 3:**
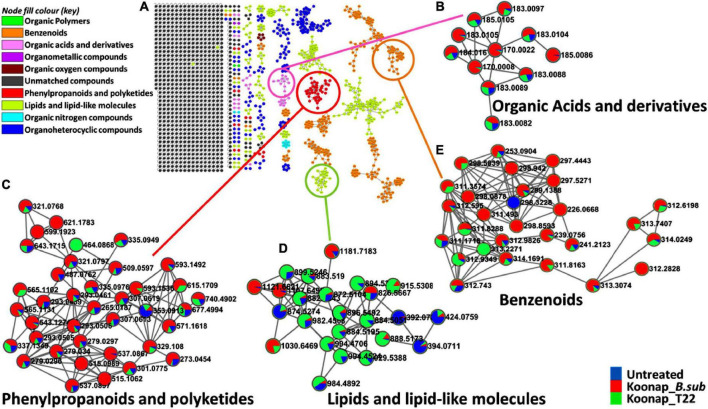
A global metabolome view of the plant growth-promoting rhizobacteria (PGPR)-treated/untreated Koonap rhizosphere showing classes of detected metabolites driving the global differences between the treated and untreated rhizosphere soils. **(A)** Molecular network of MS/MS spectra generated with MolNetEnhancer (in GNPS) giving a metabolome coverage and classes of extracted metabolites from rhizosphere soil of plant growth-promoting rhizobacteria (PGPR)-treated and untreated Koonap cultivar. Each displayed node represents a metabolite, while each cluster of pooled nodes (colored) depicts a class of chemically related and putatively annotated metabolites matched to GNPS libraries and databases. Gray nodes represent unmatched spectral data. **(B–E)** Shows clusters of organic acids and derivatives, phenylpropanoids, and polyketides, lipids and lipid-like molecules and benzenoids respectively. Each node representing a spectral data shows the differential distributions and changes in the metabolites per treatment represented by the color shading. Blue shading represents the distribution of set class of metabolites (e.g., benzenoids) in untreated samples compared to Koonap_*B. sub* (red) and Koonap_*P. alvei* T22 (green).

The exo-metabolome of rhizosphere soils is yet to be fully characterized and the presence of both microbe and plant-derived metabolites which further increases the complexity of the rhizosphere community. To study the diversity of the rhizosphere exo-metabolome and the classes of metabolites contributing to the global differences between PGPR-treated and untreated rhizosphere of wheat cultivars, molecular networking approaches were applied to mine, curate and interpret the metabolomics data as previously described (Tsugawa et al., 2015; van der Hooft et al., 2016; Quinn et al., 2017; Aron et al., 2020; Nothias et al., 2020; Othibeng et al., 2021). Figure 3A and Supplementary Figure 3 show a typical molecular network generated with tools such as feature-based molecular networking (FBMN) and MolNetEnhancer from GNPS for Koonap and Gariep, respectively. The FBMN workflow was further integrated with *in silico* annotation tools such as MS2LDA, NAP and DEREPLICATOR, generating enhanced molecular network, a MolNetEnhancer network. The computed (enhanced) network reveals molecular families, subfamilies, and subtle structural differences between family members, which then increases confidence in the class annotation (Quinn et al., 2017; Aron et al., 2020; Othibeng et al., 2021). The MN strategies allow a global annotation of the measured metabolome, revealing a number of metabolite classes including, but not limited to, benzenoids, phenylpropanoids and polyketides, organoheterocyclic compounds, organic acids, and derivatives as well as lipids and lipid-like molecules and the quantitative distribution of relative metabolites within each class as per the respective rhizosphere PGPR-treatments.

**FIGURE 4:**
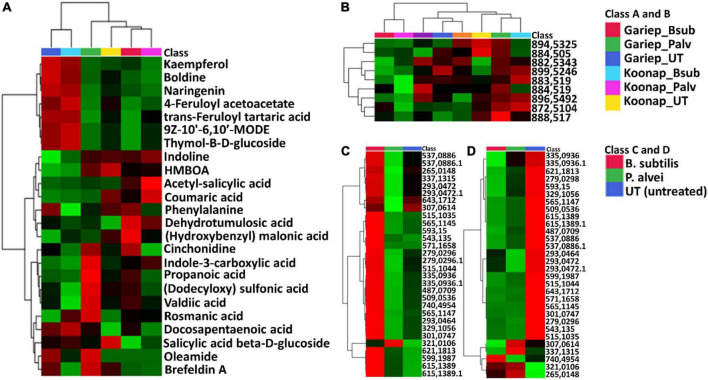
Differential quantitative profiles of annotated and non-annotated metabolites from selected main classes (phenylpropanoids, polyketides, lipids, and lipid-like molecules). The interactive heatmaps were generated from the average peak intensities of the metabolites (*n* = 9) in MetaboAnalyst. The data were median-normalized, log transformed and *Pareto*-scaled. **(A)** Shows a heatmap of putatively annotated metabolites from the rhizosphere of plant growth-promoting rhizobacteria (PGPR)-treated and untreated Koonap and Gariep cultivars. Metabolite annotations were aided by MS-DIAL and further validated through a level 2 classified Metabolomics Standard Initiative (MSI-2). Unidentified metabolites from significant classes contributing to observed variation in the plant treatment are displayed in **(B)** for lipids and lipid-like molecules. Phenylpropanoids and polyketides are shown in **(C)** and **(D)** for Koonap and Gariep, respectively.

The treatment of Koonap rhizosphere with *B. sub* resulted in the accumulation of various metabolites including organic acids and derivatives, organic oxygen compounds (e.g., salicylic acid glucoside; Figure 3B) phenylpropanoids and polyketides (e.g., kaempferol, naringenin; Figure 3C) and benzenoids (e.g., *p*-hydroxybenzyl-malonic acid; Figure 3E). In comparison *P. alvei* (T22) treatment induced the accumulation of lipids and lipid-like molecules in both Koonap and Gariep rhizosphere exo-metabolomes (Figures 3D, 4B; Supplementary Figure 3D). In contrast, phenylpropanoids and polyketides such as kaempferol and naringenin (Figures 4A,C) remained higher in untreated rhizosphere soil of Gariep, along with several organoheterocyclic compounds (Figure 4D) respectively as compared to PGPR-treated rhizosphere.

**FIGURE 5:**
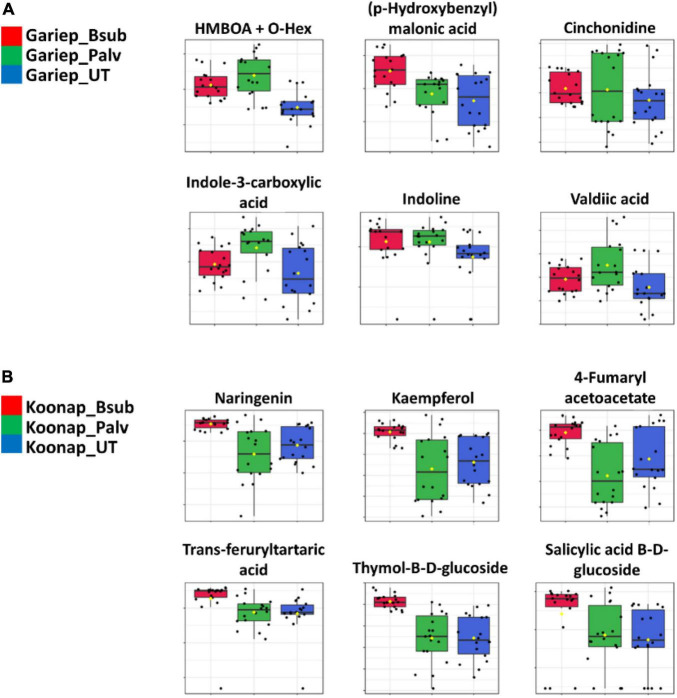
Differential quantitative profiles of increased annotated metabolites. **(A,B)** Shows the differential accumulation of selected metabolites in the Gariep and Koonap cultivars respectively. The box-and-whiskers plots reveal an increase in phenolic compounds (phenylpropanoid), organic acids and benzenoids in the rhizospheres of plant growth-promoting rhizobacteria (PGPR)-treated Gariep **(A)** and Koonap **(B)** plants. The data were median-normalized, log transformed, and *Pareto*-scaled. Red and green box-and-whiskers represent *B. sub* and *P. alvei* (T22) treatments, blue represents the untreated samples of wheat cultivars.

Furthermore, manual validation of annotated metabolites was carried out using matched fragmentation spectra. Figure 5 shows box-and-whiskers diagrams of selected increased metabolites from the PGPR-treatment in the rhizosphere of both Gariep (Figure 5A) and Koonap (Figure 5B) cultivars. Treatment with *B. sub* and *P. alvei* induced an accumulation of several metabolites, including but not limited to, indole-3-carboxylic acid, HMBOA + *O*-Hex, cinchonidine, valdiic acid, and indoline in the Gariep rhizosphere, while inducing an elevation of 4-fumarylacetoacetic acid, *trans*-feruryltartaric acid and thymol-beta-D-glucopyranoside (a bacterial metabolite) in Koonap (Figure 4A).

### Characterization of above ground (shoot) metabolite perturbations as a result of plant growth-promoting rhizobacteria-seed treatment

**FIGURE 6:**
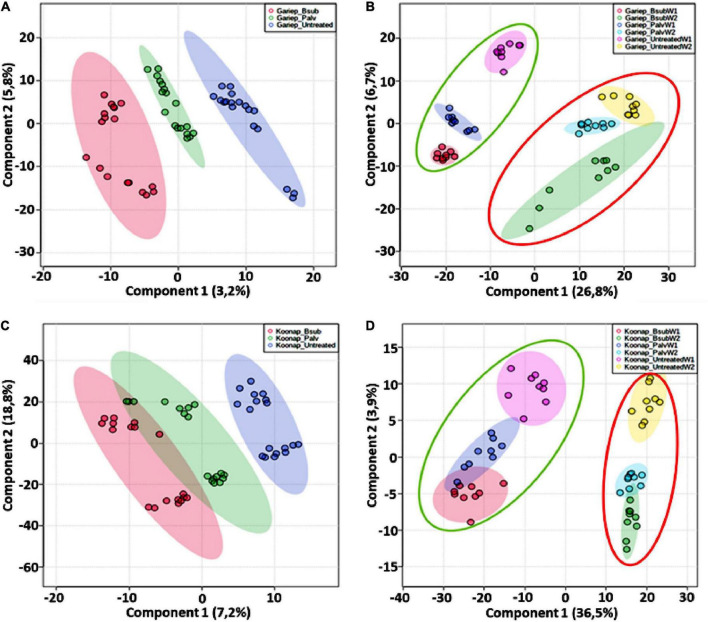
General characterization of the impact of plant growth-promoting rhizobacteria (PGPR) treatment on the overall leaf metabolome of wheat cultivars. **(A)** PLS-DA scores plot showing the global differences in the metabolite profiles from leaf extracts resulting in separation of PGPR-treated (red = Gariep_*B. sub*, green = Gariep_*P. alvei*) and untreated (blue). **(C)** PLS-DA scores plot showing the global differences in the metabolite profiles from leaf extracts resulting in separation of PGPR-treated (red = Koonap_*B. sub*, green = Koonap_*P. alvei*) and untreated (blue). **(B,C)** Shows the time-dependant clustering/separation of samples from PGPR-treated and untreated Gariep and Koonap cultivars from week 1 (green demarcation) and week 2 (red demarcation) of samples harvesting and extraction. The scores plots reveal the differential regulation of the leaf metabolome resulting from the varying rhizosphere bacterial treatments and the potential response mechanisms of the different cultivars over time. The data projected above were median-normalized, log transformed and *Pareto*-scaled in MetaboAnalyst for correlation and predictability ratings of *R*2 = 0.961 and *Q*2 = 0.578 **(A)**; *R*2 = 0.852 and *Q*2 = 0.606 **(B)**; *R*2 = 0.920 and *Q*2 = 0.418 **(C)**; and *R*2 = 0.903 and *Q*2 = 0.606 **(D)**.

Untargeted metabolomics approaches were applied to investigate changes in the leaf metabolome of PGPR-[*B. sub* and *P. alvei* (T22)]-treated of Koonap and Gariep wheat cultivars. The experimental design comprised three biological replicates and each replicate was analyzed in triplicate (i.e., three technical replicates, resulting in *n* = 9). Additionally, pooled QC samples were used to assess the stability of the analytical method and the quality of the data. PLS-DA models were generated in MetaboAnalyst to reveal the underlying structures and internal patterns of the data from a 2D perspective, reducing the multi-dimensionality and complexity of the datasets. Figure 6 illustrates the differential clustering/separation of rhizosphere PGPR-treated and untreated Gariep and Koonap leaf samples, as well as the time-course (time-dependant) changes in the metabolic profiles of samples under investigation. Figures 6A,C show group separation between the *B. sub* and *P. alvei* (T22)-treated and the untreated Gariep and Koonap samples respectively, an indication of metabolic alteration in the leaves of the plants due to PGPR treatments. Additionally, treatment of the rhizosphere with varying strains of PGPR also led to differential regulation of the wheat leaf metabolome as evidenced by the separation between Gariep_*B. sub* and Gariep_*P. alvei* (Figure 6A). Similarly, the treatment of the Koonap cultivar with *B. sub* and *P. alvei* revealed PGPR strain-specific clustering (Figure 6C), with clear overlaps suggesting shared similarities in the underlying metabolic profiles from the two treatments, while simultaneously giving insight into the differences in leaf metabolite profiles of rhizosphere treated and untreated samples. Moreover, extraction of the leaf metabolome of PGPR-treated and untreated wheat cultivars from two time points (week 1 and week 2) highlighted the time-dependant metabolic perturbations in correlation with plant development (Figure 6B-Gariep and 6D-Koonap).

**FIGURE 7:**
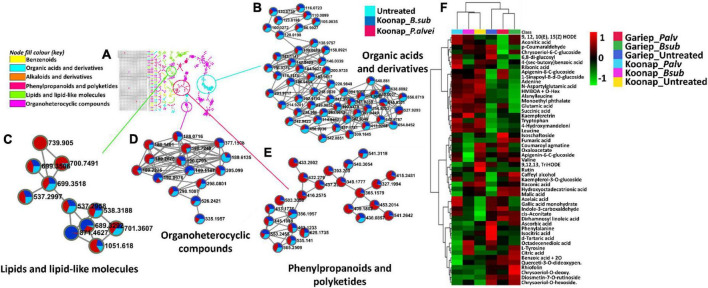
A global leaf metabolome view of the plant growth-promoting rhizobacteria (PGPR)-treated/untreated Koonap rhizosphere (Gariep-Supplementary Figure 4) showing classes and distribution of detected metabolites driving the global differences between the treated and untreated rhizosphere soils. **(A)** Molecular network of MS/MS spectra generated with MolNetEnhancer (in GNPS) giving a metabolome coverage and classes of extracted metabolites from rhizosphere soil of PGPR-treated and untreated Koonap cultivar. Each displayed node represents a metabolite, while each cluster of pooled nodes (colored) depicts a class of chemically related and putatively annotated metabolites matched to GNPS libraries and databases. Gray nodes represent unmatched metabolites. **(B–E)** Shows clusters of organic acids and derivatives, lipids and lipid-like molecules, organoheterocyclic compounds and phenylpropanoids and polyketides respectively. Each node representing a metabolite shows the differential distributions and changes in the metabolites per treatment. **(F)** Is an interactive heatmap of all putatively annotated metabolites from leaf metabolome of PGPR-treated and untreated wheat cultivar showing general up/down regulation of metabolite classes per treatment. The heatmap was generated in MetaboAnalyst from average peak intensities of median-normalized, log transformed and *Pareto*-scaled data.

Putative annotation of detected metabolites was performed at MSI level-2 annotation. Briefly, the MS spectra and empirical formulae of selected ions were compared to published data and public libraries (Supplementary Table 1). The peak intensities of annotated metabolites were further used to construct an interactive heatmap illustrating the differential accumulation and relative metabolite concentrations in the leaves of PGPR-treated and untreated rhizosphere soils of wheat cultivars (Figure 7). The heatmap reveals treatment-associated metabolite up/down-regulations, thus giving insight into the effects of each type of rhizosphere treatment on the measured metabolome of the plants. Color coordination on the heatmap represents the quantitative state of each metabolite. Red shows an up-regulation of the metabolites, black indicates an unchanged regulation while green displays decreased metabolites in response to PGPR treatment. Some increased metabolites are shown in Supplementary Figure 4.

MolNeEnhancer molecular network (Figure 7A) revealed differential distribution of metabolites in classes such as phenylpropanoids and polyketides, organoheterocyclic compounds and lipids and lipid-like molecules, showing an accumulation of some of these in PGPR-treated Koonap (Figures 7B–E). In contrast, classes of these compounds remained relatively low in the PGPR-treated Gariep cultivar as compared to untreated plants (Supplementary Figure 5). Examination of the heatmap (Figure 7F) revealed the up/down-regulation of specific metabolites based on the different treatments. For instance, Gariep_*B. sub* and Gariep_*P. alvei* rhizosphere treatments led to an up-regulation of caffeoyl alcohol, kaempferitrin, and kaempferol-3-*O*-glucoside among other metabolites that were down regulated in the untreated Gariep leaf samples. Likewise, rhizosphere treatment of Koonap with *B. sub* resulted in the up-regulation of metabolites such as azelaic acid, gallic acid monohydrate, *cis*-aconitate and indole-3-carboxyaldehyde, while *P. alvei* rhizosphere treatment induced the accumulation of citric acid, tartaric acid, octadecenedioic acid, and L-tyrosine. The up-regulation of secondary metabolites was more prevalent in the leaves of the rhizosphere-PGPR-treated Gariep cultivar compared to their untreated counterparts, in which primary metabolites were the most upregulated. In contrast, there were minor variations in the up/down-regulation of both primary and secondary metabolites in the leaves of the rhizosphere-PGPR-treated and untreated Koonap cultivar.

### Impacts of plant growth-promoting rhizobacteria-seed treatment on the primary and secondary metabolism of above ground tissue

**FIGURE 8:**
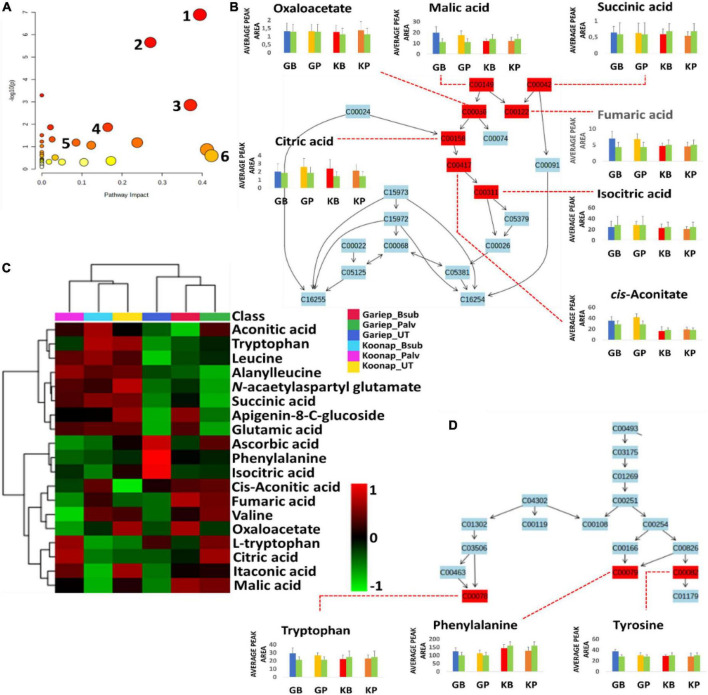
A summary of metabolic pathways analysis generated in MetPA showing significant and impactful pathways and relative quantification of altered organic acids and amino acids involved in the TCA cycle and aromatic amino acids biosynthesis. **(A)** A “metabolome view” displaying mapped metabolic pathways arranged by *p-*value for the most significant pathways on the *y*-axis and the most impactful arranged by pathway impact on the x-axis. Pathway impact values refer to the cumulative percentage from the matched metabolite nodes and the maximum importance of each pathway is 1. (1) TCA (citrate) cycle, (2) glyoxylate and dicarboxylate metabolism, (3) alanine, aspartate and glutamate metabolism, (4) phenylalanine, tyrosine, and tryptophan biosynthesis, (5) phenylpropanoid biosynthesis, and (6) phenylalanine metabolism. **(B)** A topological characterization of the TCA cycle with integrated matched intermediates (metabolites) showing altered relative quantification levels (average peak areas). **(C)** An interactive heatmap showing the differentially altered concentrations of organic and amino acids. **(D)** Topological characteristics of the aromatic amino acids biosynthesis pathway, showing the quantification levels of tryptophan, phenylalanine and tyrosine under different conditions. GB (blue) = Gariep_*B. sub*, GP (yellow) = Gariep_*P. alvei*, KB (red) = Koonap_*B. sub*, KP (orange) = Koonap_*P. alvei*. Green bars represent untreated samples for the respective cultivars.

**FIGURE 9:**
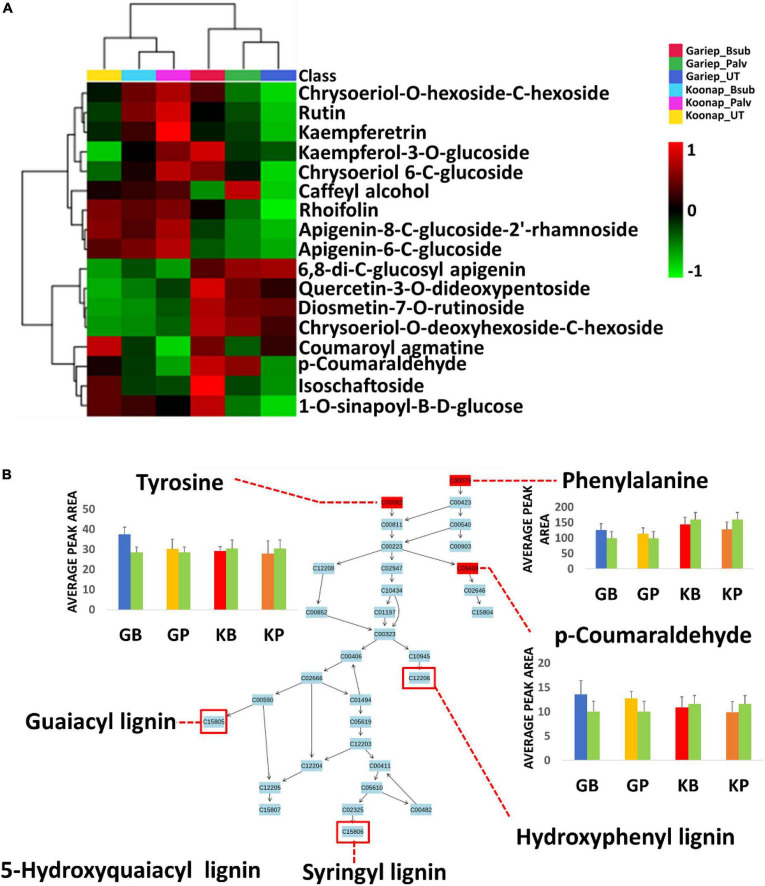
Comprehensive metabolic pathways analysis and metabolome view. **(A)** A heatmap displaying differential quantitative alterations in the concentrations of the selected phenylpropanoids. The heatmap show the up/down-regulation of some phenolic compounds in the plant growth-promoting rhizobacteria (PGPR)-treated and untreated wheat cultivars, an indication of the potential impact of PGPR treatment on secondary metabolite biosynthesis and the regulation of the secondary metabolic pathway. **(B)** Topology map of the phenylpropanoid biosynthesis pathway, indicating the integration of precursor molecules (tyrosine, phenylalanine, and tryptophan) at differentially altered quantification levels at under different conditions. GB (Blue) = Gariep_*B. sub*, GP (yellow) = Gariep_*P. alvei*, KB (red) = Koonap_*B. sub*, KP (orange) = Koonap_*P. alvei*. Green bars represent untreated samples for the respective cultivars. The end products of the phenylpropanoid biosynthesis pathways are indicated in red demarcations as lignins.

Putatively annotated metabolites (Supplementary Table 1) were used for pathway analysis on MetPA to uncover biological pathways significantly impacted by the rhizosphere PGPR treatments. The relative intensities of matched metabolites among the different pathways are illustrated in bar graphs showing the up/down-regulation of such metabolites. Matched metabolites are indicated in red (unmatched = blue) and are integrated to show the interconnectedness of the metabolites and the metabolic pathways to each other. Pathway analysis aides in visualizing metabolomics data on a broader scale taking into consideration the impact of subtle changes in the concentrations of certain metabolites from the primary or secondary metabolism of a plant. Here, the significant pathways are determined by a lower *p*-value and are found higher on the –log10(p) scale (*y*-axis), while the impactful pathways are displayed along the *x*-axis according to their pathway impact factors (Figure 8A). The most significant pathways included the TCA cycle (Figure 8B), glyoxylate, and dicarboxylate metabolism, aromatic amino acid (phenylalanine, tryptophan, and tyrosine) biosynthesis (Figure 8D) and the phenylpropanoid biosynthesis pathway (Figure 9A). The phenylalanine metabolism and alanine, aspartate and glutamate pathways were among the most impactful pathways. The results shown in Figures 8 and 9 suggest that PGPR treatment of rhizosphere soils induces metabolome perturbations in plants through a display of highly complex cellular reprograming characterized by altered metabolism spanning several metabolic pathways (Nephali et al., 2021), as discussed below.

To evaluate the impact of rhizosphere PGPR treatment on the secondary metabolism in the leaves of wheat cultivars and the potential implications on plant response and defense priming, a comprehensive metabolic pathway analysis of the phenylpropanoid biosynthesis was carried out. Here, attention was paid to the up/down-regulation (quantitative profiles) of selected putatively annotated phenolic compounds including some hydroxycinnamic acids (HCAs) and flavonoids (Figure 9). A range of flavonoids were differentially altered in the rhizosphere-PGPR-treated cultivars. For instance, rutin, kaempferol, kaempferol-3-*O* glucoside, caffeoyl alcohol, chrysoeriol-6-C-glucoside, and isoschaftoside were increased in Gariep_*B. sub* compared to the leaves of untreated Gariep samples and similar results (accumulation of flavonoids) were observed in rhizosphere-PGPR-treated Koonap cultivar leaves compared to leaves of untreated plants. Additionally, more flavonoid glycosides, including quercetin-3-*O*-dideoxypentoside, diosmetin-7-*O*-rutinoside, chrysoeriol-*O*-deoxyhexoside-*C*-hexoside and HCA derivatives such as *p*-coumaraldehyde, 1-*O*-sinapoyl-β-D-glucose and coumaroyl agmatine were relatively more abundant in the leaves of rhizosphere-PGPR-treated wheat cultivars than their untreated counterparts.

## Discussion

The chemistry of the rhizosphere is a complex mixture of compounds from plant root exudates and secretions of microbial breakdown compounds. These compounds drive the phenomenology of plant–microbe interactions in the rhizosphere as well as inter-rhizosphere bacterial intercommunications (Mhlongo et al., 2018). However, the mechanisms and dynamics by which these chemical interactions occur remain enigmatic; and this could be because the chemical space of rhizosphere soils (rhizosphere exo-metabolome) has not yet been fully characterized. The comprehensive characterization and profiling of the rhizosphere can thus shed light into the varied mechanisms by which plants communicate with and are impacted by interactions from their rhizosphere inhabiting microbial neighbors.

Major experimentation on the elucidation of the chemical space of the rhizosphere rely on hydroponics and sterile growth systems (Pétriacq et al., 2017), which are suitable for the uninterrupted and direct quantification of plant root exudates. However, these approaches do not represent the natural dynamics of the rhizosphere in which plants are in constant interaction with rhizosphere-inhabiting microorganisms and therefore do not account for the metabolites produced and secreted from these microbes thus limiting the exploration of the chemical diversity of the rhizosphere (Mashabela et al., 2022a). In this study, we performed a global metabolite profiling of rhizosphere soils of PGPR-treated (seed-bio-primed) wheat cultivars to reveal classes of metabolites creating the much elusive chemical diversity of the rhizosphere and investigate the effects of seed bio-priming on the above ground (shoot) metabolism of PGPR-treated cultivars in a bottom-up metabolomics study.

### Chemical diversity and classification of metabolites from rhizosphere soils of plant growth-promoting rhizobacteria-treated (seed bio-primed) wheat cultivars

An untargeted UHPLC-Q-TOF-MS analysis of rhizosphere soils of PGPR-bio-primed wheat cultivars, combined with classical and FBMN tools revealed the chemistry and chemical diversity of the rhizosphere. The metabolomics approach led to the detected classes of primary and secondary metabolites, including organic acids and derivatives, organoheterocyclic compounds, lipids and lipid-like molecules, phenylpropanoids and polyketides as well as benzenoids and derivatives as major metabolite classes in the rhizosphere. The detected metabolites varied in treatment- and timepoint-based qualitative and quantitative distribution across the rhizosphere exo-metabolome.

Benzenoids and derivatives accounted for most compounds detected in the rhizosphere, followed by lipids and lipid-like molecules and organoheterocyclic compounds. Benzenoids are a class of specialized aromatic metabolites that are ubiquitous in the plant kingdom generally occurring as volatile organic compounds (VOCs). These compounds are also rapidly produced and secreted by microorganisms and play an important role in plant defense, stress response and mediation at the plant–microbe interface (Junker and Tholl, 2013; Misztal et al., 2015; Lackus et al., 2021). Their roles in belowground (rhizosphere) plant–plant and plant–microbe communications have only started to emerge recently (Schenkel et al., 2018). For instance, 4-(*p*)-hydroxymandelonitrile, an L-tyrosine-derived benzenoid identified in this study, is a compound involved in the biosynthesis of dhurrin, a member of bioactive cyanogenic glycosides (CGs) and hydrogen cyanides (HCNs) found in many cultivated cereal crops including barley (*Hordeum vulgare*), sorghum (*Sorghum bicolor* (L.) Moench) and wheat (*Triticum aestivum*) (Kojima et al., 1979; Erb et al., 1981; Jones, 1998; Nielsen et al., 2002; Nielsen et al., 2016).

Cyanogenic glycosides and HCNs are important specialized natural toxins with roles in plant defense against herbivores, insects, and pathogens (Kytidou et al., 2020; Nyirenda, 2020), but are also found in some species of fungi and bacteria and could be involved in biocontrol mechanisms of these microbes (Kytidou et al., 2020; Nyirenda, 2020). According to a study by De Brida et al. (2018), oat, wheat and sorghum are recommended for crop rotation systems due to their ability to produce HCNs for nematode and pathogen suppression, as evidenced by their suppression of the root-knot nematode *Meloidogyne enterolobii*. In this regard, 4-(*p*)-hydroxymandelonitrile could be released through exudation by the plant into the rhizosphere as a defense metabolite against potential pathogenic attacks, or a defense signaling molecule from the PGPR-treatment to aid in plant defense response.

Derivatives of benzenoids [i.e., benzoxazinoids (BXs), e.g., 4-acetyl-2(3H)-benzoxazolone (ABOA), HMBOA (2-hydroxy-7-methoxy-2H-1,4-benzoxazin-3(4H)-one) + O-Hex] were also identified in this study. BXs [a family of approximately 20 compounds sharing the HBOA (2-hydroxy-2H-1,4-benzoxazin-3(4H)-one skeleton)] are considered agriculturally relevant specialized plant metabolites and important factors in plant–microbe interactions, particularly parasite recognition in cereals (Poaceae) such as maize (*Zea mays*), wheat (Triticum spp.), rye (*Secale cereale*) (Steffens et al., 2017; Zhou et al., 2018). These phytochemicals are ubiquitous in grasses, particularly in cereal crops as general defense metabolites and display allelopathic, antifeedant, insecticidal and antimicrobial activities (Wouters et al., 2016a,b, 2018; Niculaes et al., 2018; Mwenda et al., 2021).

Detection of ABOA and HMBOA + O-Hex in the rhizosphere soils of wheat cultivars correlates with findings by Mwenda et al. (2021), in which the authors detected fifteen unique BXs and their derivatives in roots, shoots, rhizoplane, and rhizosphere soil extracts of wheat and rye. The study revealed elevated levels of some of the most commonly occurring BXs [MBOA, HMBOA, and HMBOA-Glc (hex)] in wheat tissues, particularly in the roots and rhizoplane at vegetative growth stages. In the current study, ABOA and HMBOA+ O-Hex were found more abundantly in rhizosphere soils of PGPR-treated Gariep cultivars compared to the control, while untreated Koonap cultivars displayed higher content of the BXs relative to their PGPG-treated counterparts, pointing to cultivar-specific exudation and rhizosphere metabolite accumulation. Cultivar-specific root exudation of metabolites can have critical ecological impacts on the rhizosphere microbial community and the physical properties of the soil. Moreover, BXs have been shown to act as chemoattractant metabolites. Chemotaxis assays by Neal et al. (2012) showed the mobility of *Pseudomonas putida* toward DIMBOA [2,4-dihydroxy-7-methoxy-2H-1,4-benzoxazin-3(4H)-one], in the rhizosphere of DIMBOA-producing maize cultivars and this could be an indicator of the mechanisms of plant–microbe interactions at the biochemical level if investigated further.

Plant root exudation has been one way to actively transform and modify the conditions of the rhizosphere, additionally, root exudates are generally considered as the first line of communication between the plant and resident microorganisms in the rhizosphere (Oburger et al., 2009; Wu et al., 2017). Among these exudates, organic acids are the most common and have drawn much attention from researchers due to their versatile profile as functional rhizosphere metabolites (Tuason and Arocena, 2009). The rhizosphere of the wheat cultivars showed an abundance of organic acids irrespective of treatment, i.e., PGPR-treated Koonap and untreated Gariep cultivars showed similar exo-metabolite profiles of organic acids, indicating the constitutive nature of these compounds as root exudates. The class of organic acids identified in the current study constituted a plethora of low molecular weight organic acids (LMWOAs, MW < 500). These compounds are primary exudates of plant roots and can be synthesized by microorganisms (Tuason and Arocena, 2009). LMWOAs are strongly associated with the solubilization of essential nutrients, stimulation of microbial growth and the detoxification of toxic metals (e.g., Al^3+^) as well as driving rhizosphere bacterial chemotaxis (Nwoke et al., 2008; Menezes-Blackburn et al., 2016; Jiang et al., 2017; Macias-Benitez et al., 2020; Mashabela et al., 2022b).

The OA 4-fumarylacetoacetate that was identified in this study, is an essential intermediate metabolite in the degradation pathway of tyrosine, phenylalanine (also identified in this study) and other aromatic compounds by PGPR such as *Pseudomonas putida* (Arias-Barrau et al., 2004; Singh et al., 2018). Catalyzed by the enzyme fumarylacetoacetate hydrolase, 4-fumarylacetoacetate is broken down to fumarate and acetoacetate. This revelation could point to the presence of 4-fumarylacetoacetate in the rhizosphere soils of the wheat cultivars particularly in Koonap cultivars treated with *B. subtilis* and *P. alvei* as an intermediate of PGPR-mediated phenylalanine catabolism. According to Singh et al. (2018), aromatic amino acids are some of the major components of root exudates and PGPR utilize the catabolized aromatic compounds from plants as one strategy to confer selective advantage in the rhizosphere. Additionally, the PGPR *Pseudomonas putida* (strain KT2440) uses aromatic compounds such as benzoates, phenyls and tyrosine as carbon sources (Jiménez et al., 2002; Singh et al., 2018). In addition to aromatic amino acids, rhizosphere inhabiting microorganisms also metabolize organic acids to use as a source of carbon and energy (Macias-Benitez et al., 2020). Furthermore, organic acids (gluconate, citrate, lactate, and succinate) have been reported to reduce the soil pH through phosphorous solubilization in the rhizosphere, this could be especially important in reducing the effects of metal toxicity in acidic soils thus promoting tolerance in naïve plants (Martínez-Viveros et al., 2010).

Indoles and indole derivatives are a class of organoheterocyclic compounds that are constitutive in nature (Kumar et al., 2021). An indole derived phytohormone, indole-3-carboxaldehyde (ICA), was detected in higher concentrations from the *B. subtilis* and *P. alvei*-treated rhizosphere soils of Gariep as well as *P. alvei*-treated rhizosphere soils of Koonap wheat cultivars as compared to the untreated cultivars. These findings suggest the production of IAA and consequently ICA by both *B. subtilis* and *P. alvei* through tryptophan metabolism, which could further be secreted into the rhizosphere to enhance plant growth, development and possibly defense response. A study by Talboys et al. (2014) reported the production of IAA from species of *Bacillus amyloliquefaciens* (strain FZB42), leading to increased root development following a dressing on the seeds of *Triticum aestivum*. High auxin production potential was also recorded in the PGPR *B. subtilis* (Mt3b) isolated from rhizosphere of *Malvastrum tricuspidatum* (Wagi and Ahmed, 2019). A recent comprehensive study by Kiruthika and Arunkumar (2020) showed that *B. subtilis* had the potential to produce IAA and subsequently ICA, resulting in improved root and shoot length as well as dry weight and overall plant growth. Additionally, *Paenibacillus* species have been reported to produce IAA in contribution to plant growth (Xu and Kim, 2014; Grady et al., 2016). The studies above proved bacterial IAA to be an efficient metabolite in the mediation of plant growth and improvement. Furthermore, the presence of ICA in the rhizosphere soils of PGPR-treated wheat cultivar could be a proof of principle of the production of other phytohormones (Suliasih, and Widawati, 2020).

Indoles are known to play important roles as signal cues and performance enhancers in eukaryotic life forms and are well documented for their roles as inter-species and inter-kingdom signaling (Tomberlin et al., 2017; Kumar et al., 2021). The biosynthesis of indoles and indole derivatives in most multispecies communities has been attributed to microbial metabolism, with functions in resistance or biofilm formation (Lee et al., 2007) and the oxidized forms of indoles in the latter function could help mediate rhizosphere colonization by allowing microbial attachment to plant roots (Lee et al., 2015). Indole-3-carboxaldehyde (ICA) is a product of bacterial tryptophan metabolism. It is produced from the decarboxylation of indole-3-acetic acid (IAA), the most commonly occurring auxin (plant hormone) produced by terrestrial plants and is responsible for plant development through a variety of cellular mechanisms including alteration of cell orientation, organ development, fertility and cell elongation (Fu et al., 2015; Labeeuw et al., 2016). In addition to IAA biosynthesis, plants are the main source of tryptophan in soils in the form of root exudates (Etesami et al., 2008), which is used by bacteria to produce IAA through several tryptophan-dependent pathways (reviewed by Spaepen et al., 2007; Fu et al., 2015; Shameer and Prasad, 2018).

The exo-metabolome of the rhizosphere soils also showed profiles of lipid and lipid-like molecules as well as phenylpropanoids and polyketides. Lipids are a broad class of complex heterogenous molecules that share common properties of hydrophobicity (Burdge and Calder, 2015). These molecules are generally made up of repeating units of fatty acids with varying structural orientations which further contribute to their metabolism and functional differences (Burdge and Calder, 2015; Pateiro et al., 2019). In the rhizosphere, microbe recognition by plants occurs at the plasma membrane which acts as the interface, followed by a downstream signaling cascade and the appropriate response mechanisms based on the environmental condition (Macabuhay et al., 2021). Lipids in various forms, such as fatty acids are among the compounds enriching the rhizosphere chemical space and modulators of plant–microbe interactions (Macabuhay et al., 2021). Additionally, Bi et al. (2021) detected fatty acids in the rhizosphere soil of five plant species and reported their effects on the adaptation of microbial taxa to arid environments, indicating the use of fatty acids by plants to modulate the rhizosphere microbial community.

The analysis of mucilage secreted from roots and seeds of maize, lupin, and wheat found a lipid composition of 3.1% composed of fatty acids (Nazari, 2021), which were reported to function as lubricants for root penetration through the soil and help in water uptake from the rhizosphere by altering the soil hydraulic properties. Furthermore, several studies have reported fatty acids as inhibitors of pathogen growth, reduce the occurrence of crop diseases, improve rhizosphere conditions and plant growth (Liu et al., 2008; Liu et al., 2012; Ma et al., 2021). Ma et al. (2021) found that the application of palmitic acid (PA) on the rhizosphere of watermelon resulted in the reduction of in the disease severity of fusarium wilt and promoted plant growth. The detection of fatty acids such as methyl (9Z)-10′-oxo-6,10′-diapo-6-carotenoate and oleamide in the current study could point to the function of lipids (and lipid-like molecules) in the rhizosphere as discussed above.

According to a study by Narasimhan et al. (2003), auxotrophic microbes grow and proliferate at a slower rate than phenylpropanoid-utilizing microbes. The authors reported better colonization of the rhizosphere of phenylpropanoid-producing and secreting Arabidopsis mutants. Additionally, microbes interacting with these mutants were found to be more competitive and grow 100-fold better than their auxotrophic counterparts, in part due to their phenylpropanoid-metabolizing capabilities for carbon nutrition. Several bacterial species have evolved to produce secondary metabolites with various biological functions (Gokulan et al., 2014). However, major classes of biologically active compounds such as phenylpropanoids and flavonoids are largely absent from bacteria (Moore et al., 2002; Kaneko et al., 2003; Gokulan et al., 2014). On the other hand, Narasimhan et al. (2003) showed that phenylpropanoids constitute approximately 84% of secondary metabolites exuded from Arabidopsis roots. The current study reports on the rhizosphere exo-metabolome profile inclusive of phenylpropanoids and relative polyketides. Based on the above information, it can be speculated that plants exude these secondary metabolites into the rhizosphere to serve as chemotaxis strategies for the recruitment of beneficial rhizosphere-inhabiting microbes to shape the rhizosphere microbial community and facilitate plant–microbe interactions (Tilston et al., 2014; Zuluaga et al., 2021). In support of this speculation, it was observed that the rhizosphere of untreated Gariep cultivars contained elevated concentrations of phenylpropanoids compared to their PGPR- (*B. sub* and *P. alvei*)-treated counterparts. In this regard, *B. sub-*treated rhizosphere soils of the Koonap cultivar had an abundance of phenylpropanoids, indicating a treatment-independent exudation of these secondary metabolites into the rhizosphere.

### Metabolite reprograming of aboveground tissue of plant growth-promoting rhizobacteria-treated (seed bio-primed) wheat cultivars

Metabolite reprograming or perturbations (increases or decreases of certain metabolite) in plants occur at different growth stages to account for and facilitate the metabolic requirements for growth and regulation of plant metabolism during the plant’s developmental phases. Unsurprisingly, the reprograming of a plant’s metabolic status is expected due to constant interactions with factors from the immediate environment, such as biotic and/or abiotic stress agents, climatic conditions, or in communication with neighboring plants. As such, metabolic alterations spanning several metabolite classes are required for environmental adaptation, stress response, and signal transduction. In the current study, PGPR-induced metabolic perturbations spanned classes such as phenolics (flavonoids and HCAs), lipids, organic acids, and amino acids. These metabolic alterations could possibly point to certain metabolites as markers for PGPR-induced plant priming for environmental adaptations against biotic and abiotic stress as well as plant growth and development.

### Time-dependant and plant growth-promoting rhizobacteria-induced global metabolic reprograming of bio-primed seed wheat cultivars as revealed by molecular networking tools

The application of molecular networking tools [Figure 7 (Koonap) and Supplementary Figure 5 (Gariep)] revealed metabolic reprograming in the leaves of rhizosphere PGPR-treated wheat cultivars. PGPR-induced metabolic perturbations spanned classes of metabolites such as phenolics (flavonoids and HCAs), lipids, organic acids, and [annotated] amino acids. These metabolic alterations impacted important pathways facilitating the plant’s primary and secondary metabolism including the TCA (citrate) cycle, glyoxylate and dicarboxylate metabolism, alanine, aspartate, and glutamate metabolism, phenylalanine, tyrosine and tryptophan biosynthesis, phenylpropanoid biosynthesis, and phenylalanine metabolism (Figure 8A). Figures 6A,B demonstrate the differential metabolite profiles from the leaves of wheat cultivars as the result of PGPR treatments. From this observation it is safe to assume that the PGPR seed bio-priming induce metabolic alterations of above ground tissues of treated plants. A time-course metabolic reprograming of PGPR-treated plants was reported by Mhlongo et al. (2020) and the study revealed the differential reprograming of molecules identified as potential markers for PGPR priming in treated tomato plants. The current study showed the time-dependant metabolite perturbations observed in both PGPR-treated and untreated wheat cultivars and this serves as indication for the apparent metabolic changes occurring in plants during growth and development.

### Differential changes in primary and secondary metabolism of plant growth-promoting rhizobacteriaseed bio-primed wheat cultivars

The evaluation of the interactive heatmap (Figure 8C) revealed significant metabolite features that differentiate PGPR-primed and naïve Gariep cultivars. PGPR-treatment induced changes in the TCA cycle intermediates (organic acids) such as citrate, malate, oxaloacetate, succinate, fumarate, and *cis*-aconitate and amino acids (phenylalanine, tryptophan, and tyrosine) content of primed cultivars compared to their untreated counterparts. An increase in these compounds showed the redirection of primary metabolic activity to energy and aromatic amino acid biosynthesis. Considering that the TCA cycle feeds into the amino acid biosynthetic pathway, this observation suggests that the alteration in the primary metabolism of PGPR-primed Gariep cultivars was channeled into defense-related proteins and metabolic pathways as further discussed below (Gamir et al., 2014; Pastor et al., 2014; Tugizimana et al., 2019). Organic acids play a crucial role in plant metabolism by regulating numerous metabolic processes. OA fluxes mediate signal transduction processes through contributions to ROS (reactive oxygen species) and RNS (reactive nitrogen species) generation, and also facilitate the redox state of cells and maintain osmoregulation (Igamberdiev and Bykova, 2018). In photosynthesis, OAs such as malate and citrate regulate the transport of redox equivalents across cellular compartments (chloroplasts and the cytosol) to support redox balance and amino acid biosynthesis respectively (Igamberdiev and Eprintsev, 2016; Igamberdiev and Bykova, 2018). Furthermore, oxaloacetate facilitates CO_2_ fixation in the C4 photosynthetic pathway (Ludwig, 2016).

According to a study by Nephali et al. (2021), these TCA intermediates can provide alternative sources of carbon leading to a well-maintained photosynthetic machinery which can help plants in stress-drought-tolerance. Additionally, OAs such as citric acid have been reported to increase in leaf tissue following infection of tomato plants with *Phytophthora capsici* (Mhlongo et al., 2020). This phenomenon was speculated to drive the OA-mediated biosynthesis of fatty acids, which serves in lipid signaling and the formation of lipid peroxyl radicals or cell membrane destruction following pathogenic infection, thus limiting disease progression in infected plants (Zeiss et al., 2018; Mhlongo et al., 2020). The accumulation of OAs in the PGPR-treated Gariep cultivar could reflect the primed state of the plant, which is beneficial for a faster and more robust defense response in the case of pathogenic infections or display stronger stress tolerance.

Amino acid metabolism and biosynthesis were also impacted by PGPR-treatments, resulting in the accumulation of aromatic amino acids in treated compared to the untreated Gariep cultivar. Metabolic reprograming in the leaves of *Cicer arietinum* L. was observed after treatment with *Bacillus* spp. PGPR (Khan et al., 2019). The study reported alterations in the amino acid metabolism and biosynthesis, among other metabolic pathways, following PGPR treatments. The authors inferred those changes in the metabolic sphere could influence physiological parameters that may confer plant protection from environmental stress. Amino acids are primary precursor molecules in protein synthesis. In addition, they serve as pivotal building blocks for the biosynthesis of secondary metabolites involved in plant defense or resistance against biotic and abiotic stress. Aromatic amino acids are incorporated into secondary metabolite biosynthesis through the shikimate pathway. This pathway is seen as an important gateway to plant defense, and it is generally activated under stress conditions to produce aromatic amino acids such as tryptophan, phenylalanine and tyrosine which form precursors of secondary metabolite biosynthesis and play important roles in plant–pathogen interactions and plant priming (Pott et al., 2019; Jan et al., 2021).

Plant growth-promoting rhizobacteria-treatment was found to differentially regulate phenylalanine concentrations in leaves of the wheat cultivars. Phenylalanine is deaminated to cinnamic acid at the initial stages of the phenylpropanoid pathway, where further metabolism leads to the production of monolignols such as gualacyl lignin, *S*-hydroxygualacyl lignin, syringyl lignin, and hydroxyphenyl lignin (Figure 9B), which enhance cell wall lignification for mechanical resistance against pathogens (Mhlongo et al., 2020). Similar to phenylalanine, tyrosine is a product of the shikimate pathway that functions as a precursor of phenol biosynthesis which are essential for plant defense responses (Tugizimana et al., 2019; Mhlongo et al., 2020). On the other hand, tryptophan is a significant metabolite for plant signaling and defense-related plant responses. It is a precursor of indole-3-acetic acid (IAA), the most common auxin produced by plants which influences a variety of cellular mechanisms, including stress signaling and plant root and shoot development (Prusty et al., 2004; Fu et al., 2015; Labeeuw et al., 2016).

Studies have reported that the priming phase in plants is characterized by alterations in primary metabolism. Tugizimana et al. (2019) reported the accumulation of tryptophan and tyrosine in primed *Sorghum bicolor* plants following infection with *Colletotrichum sublineolum* compared to the infected naïve plants. Similar results were reported by Ward et al. (2010) where treatment of Arabidopsis with virulent and avirulent pathogens (*Pseudomonas syringae* pv. tomato DC3000) led to differential accumulation of amino acids discussed above, highlighting the functional roles of these amino acids in plant defense and immune response. Furthermore, tyrosine is a precursor molecule to the cyanogenic plant defense compound dhurrin. Surprisingly, the altered levels of OAs and amino acids, i.e., the accumulation of these compounds, were not observed in the PGPR-treated Koonap cultivar. On the contrary, levels of OAs and amino acids stayed relatively low in the treated Koonap cultivar as compared to the naïve (untreated) plants. As reported by Mashabela et al. (2022b), the Koonap cultivar is a wheat cultivar displaying resistance to biotic (*Pst*) and abiotic (Al^3+^ toxicity) stress conferred mainly by the generally higher levels of phenolic compounds and other secondary metabolites. Therefore, a redirection of amino acid biosynthesis for energy metabolism and a PGPR-induced OAs release from the plants as root exudates (Figure 3B) is possible. Furthermore, the correlation of metabolic reprograming and PGPR treatment could be cultivar specific (Killiny and Hijaz, 2016).

Further quantitative evaluation of the putatively annotated metabolites reveals potential biomarkers for plant response to PGPR treatment and plant priming from significant reprograming of phenolic metabolite profiles (Figure 9A). A positive correlation was observed between PGPR treatment and the accumulation of phenolic compounds in both the Gariep and Koonap cultivars. Rhizosphere PGPR treatments showed *B. sub*- and *P. alvei*-induced reprograming of defense-related flavonoids such as rutin, isoschaftoside, quercetin-3-*O*-dideoxypentoside, rhoifolin, and apigenin-8-C-glucoside-2′-rhamnoside in the Gariep cultivar. On the other hand, the accumulation of apigenin-6-*C*-glucoside, rutin, kaempferol-3-*O*-glucoside and diosmetin-7-*O*-rutinoside among others were observed in both the *B. sub* and *P. alvei*-treated Koonap cultivar. The alterations in the secondary metabolism (phenolic content) of PGPR-treated wheat cultivars could serve as a priming mechanism in plants to deploy a more robust, stronger and lasting defense response upon pathogen recognition compared to naïve plants. The observed metabolic reconfiguration could also be a gateway to PGPR-mediated ISR as reported by Carlson et al. (2019) who observed the up-regulation of defense-related metabolites in *P. alvei*-primed *S. bicolor* plants responding to *F. pseudograminearum* infection. Similarly, changes in the secondary metabolism constituting the accumulation of chemical and structural defense metabolites such as flavonoids, hydroxycinnamates, stilbenoids, coumarins, and lignins was reported in *Brassica oleracea* species treated with root-associated strains of three *Paraburkholderia* species. The observed metabolic alterations were associated with plant growth and induced resistance against the bacterial leaf pathogen *Xanthomonas campestris* (Jeon et al., 2021). Altogether, the biochemical events elucidated above show the diversity and complexity of the rhizosphere as well as the far-reaching impacts of changes in the chemical space of the rhizosphere on the distal aboveground tissue of due to PGPR inoculation.

## Conclusion and future perspectives

In this study, the diverse and complex metabolome of rhizosphere soils from PGPR-treated wheat cultivars was investigated. PGPR-treatment induced metabolic changes in both below (rhizosphere) and above ground chemical spaces of inoculated plants spanning classes of metabolites including, but not limited to, phenylpropanoids, organic acids, lipids, organoheterocyclic compounds, and benzenoids associated with plant–microbe communications, chemotaxis, biocontrol, and plant growth and development. As such, understanding the intricate biochemical diversity and complexity of the rhizosphere could shed light into the complex chemical intercommunications between plants and microbes. These efforts may lead to a better understanding of the essential microbe-produced metabolites required for improvement of plant physiological processes, further allowing for effective PGPR-mediated metabolic engineering for sustainable agriculture. However, minor challenges remain, and these include the near impossible task of determining the origins of each metabolite (microbe- or plant-produced) in the rhizosphere, the knowledge of which could help with the identification of microbe-specific plant beneficial metabolites, or plant-specific metabolites essential for microbial accumulation and survival in the rhizosphere. Future studies can include the application of isotope labeling technologies to track compound metabolism in both plants and microbe that could help distinguish between plant-exuded and microbe-secreted metabolites in the rhizosphere. Additionally, targeted metabolomics studies can be applied to quantitatively elucidate specified metabolites responsible for direct or indirect plant–microbe interactions. Furthermore, LC-MS can be limited in the detection of the complete metabolome of biological systems due to the variation in the physicochemical properties of the metabolites. As such, additional analytical techniques can be utilized including gas chromatography mass spectrometry (GC-MS), which offers better performance for detection of other metabolites, including volatile compounds. Additionally, further elucidation of detected metabolites can be done on NMR, which allows users to obtain rich structural information from the vibrations of the molecules in their natural environment for a wider coverage of the metabolome.

## Data availability statement

The raw data supporting the conclusions of this article will be made available by the authors, without undue reservation.

## Author contributions

MIM conceived the project and guided the writing of the manuscript. MDM and PS carried out the experimental work and data analysis and interpretation. MDM performed an initial literature search and writing—original draft preparation. MIM, LP, FT, and ID did writing—review and editing. MIM did funding acquisition. All authors read and agreed to the published version of the manuscript.

## Footnotes

1
https://www.metaboanalyst.ca/

2
https://dnp.chemnetbase.com/

3
https://pubchem.ncbi.nlm.nih.gov/

4
https://www.chemspider.com/

5
https://www.reifycs.com/AbfConverter/

6
https://gnps.ucsd.edu/

7
http://prime.psc.riken.jp/compms/msdial/main.html#MSP

8
www.metaboanalyst.ca
