# Supplementary material for: Untargeted metabolite profiling to elucidate rhizosphere and leaf metabolome changes of wheat cultivars (Triticum aestivum L.) treated with the plant growth-promoting rhizobacteria Paenibacillus alvei (T22) and Bacillus subtilis
Source: Front Microbiol. 2022 Aug 25;13:971836. doi: 10.3389/fmicb.2022.971836 (PMC9453603; doi:10.3389/fmicb.2022.971836)
Supplement: Supplementary file 1 [file Data_Sheet_1.pdf]

Untargeted metabolite profiling to elucidate rhizosphere and leaf metabolome changes of wheat cultivars (*Triticum aestivum* L.) treated with the PGPR *Paenibacillus alvei* (T22) and *Bacillus subtilis*

Manamele D. Mashabela[1], Fidele Tugizimana[1, 2], Paul A. Steenkamp[1], Lizelle A. Piater[1], Ian A. Dubery[1], Msizi I. Mhlongo[1*]

[1]Research Centre for Plant Metabolomics, Department of Biochemistry, University of Johannesburg, Johannesburg, South Africa

[2]International Research and Development Division, Omnia Group, Ltd., Johannesburg, South Africa

***Correspondence:**
Msizi I. Mhlongo
mmhlongo@uj.ac.za

Supplementary material

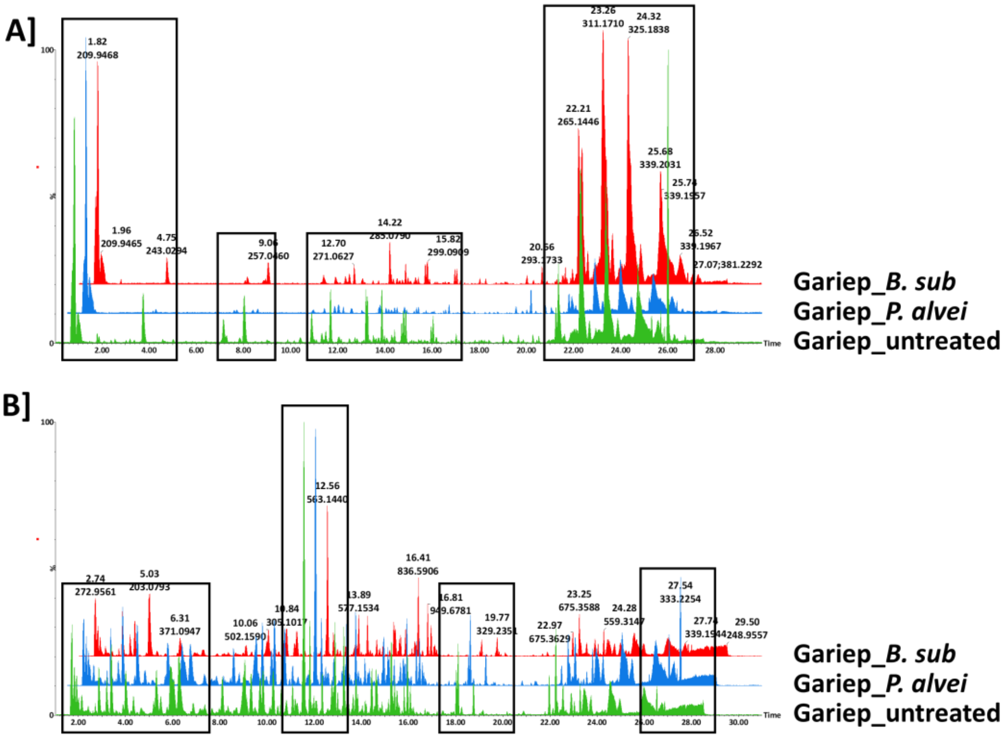

Figure S1: UHPLC-MS detection of primary and secondary metabolites from rhizosphere and leaf extracts. The figure gives a primary comparison of the metabolite profiles of PGPR-treated (red = Gariep_*B. sub* and blue = Gariep_*P. alvei*) against untreated Gariep cultivar (green) from rhizosphere **(A)** and leaf **(B)** extracts. Extracts were separated on an HSS T3 reverse-phase column and presented above are ion chromatograms from the ESI negative ionisation mode. Leaf extracts resulted in a higher volume of detected ions as compared to the rhizosphere exo-metabolome, while inter-treatment variations in metabolite profiles were observed as shown in black boxes.

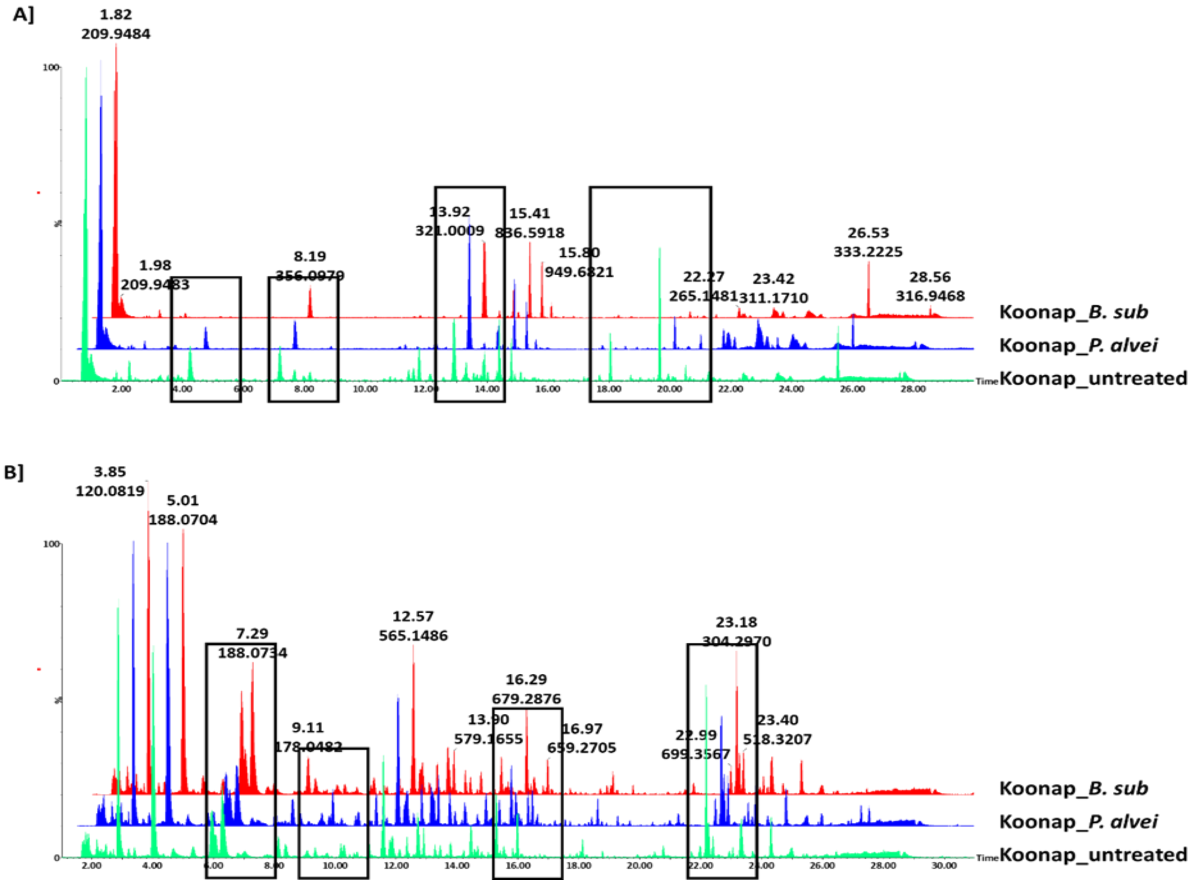

Figure S2: UHPLC-MS detection. The figure gives a primary comparison of the metabolite profiles of PGPR-treated (red = Koonap_*B. sub* and blue = Koonap_*P. alvei*) against untreated Koonap cultivar (green) from rhizosphere (**A**) and leaf (**B**) extracts. Extracts were separated on an HSS T3 reverse-phase column and presented above are ion chromatograms from an ESI negative ionisation mode. Leaf extracts resulted in a higher volume of detected ions as compared to the rhizosphere exo-metabolome, while inter-treatment variations in metabolite profiles were observed as shown in black boxes.

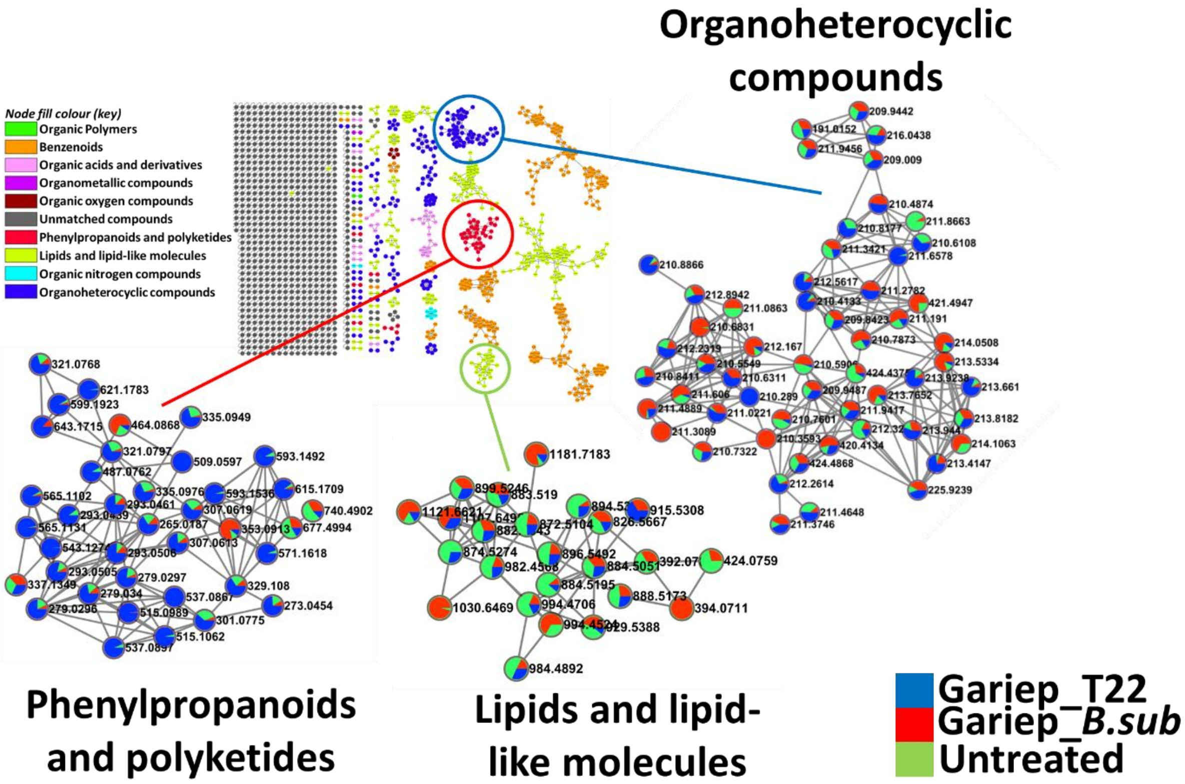

Figure S3: A global metabolome view of the PGPR-treated/untreated Gariep rhizosphere showing classes of detected metabolites driving the global differences between the treated and untreated rhizosphere soils. **(A)** Molecular network of MS/MS spectra generated with MolNetEnhancer (in GNPS) giving a metabolome coverage and classes of extracted metabolites from rhizosphere soil of PGPR-treated and untreated Gariep cultivar. Each displayed node represents a metabolite, while each cluster of pooled nodes (coloured) depicts a class of chemically related and putatively annotated metabolites matched to GNPS libraries and databases. Grey nodes represent unmatched spectral data. **B, C, D** and **E** show clusters of organic acids and derivatives, phenylpropanoids and polyketides, lipids and lipid-like molecules and benzenoids respectively. Each node representing a metabolite shows the differential distributions and changes in the metabolites per treatment.

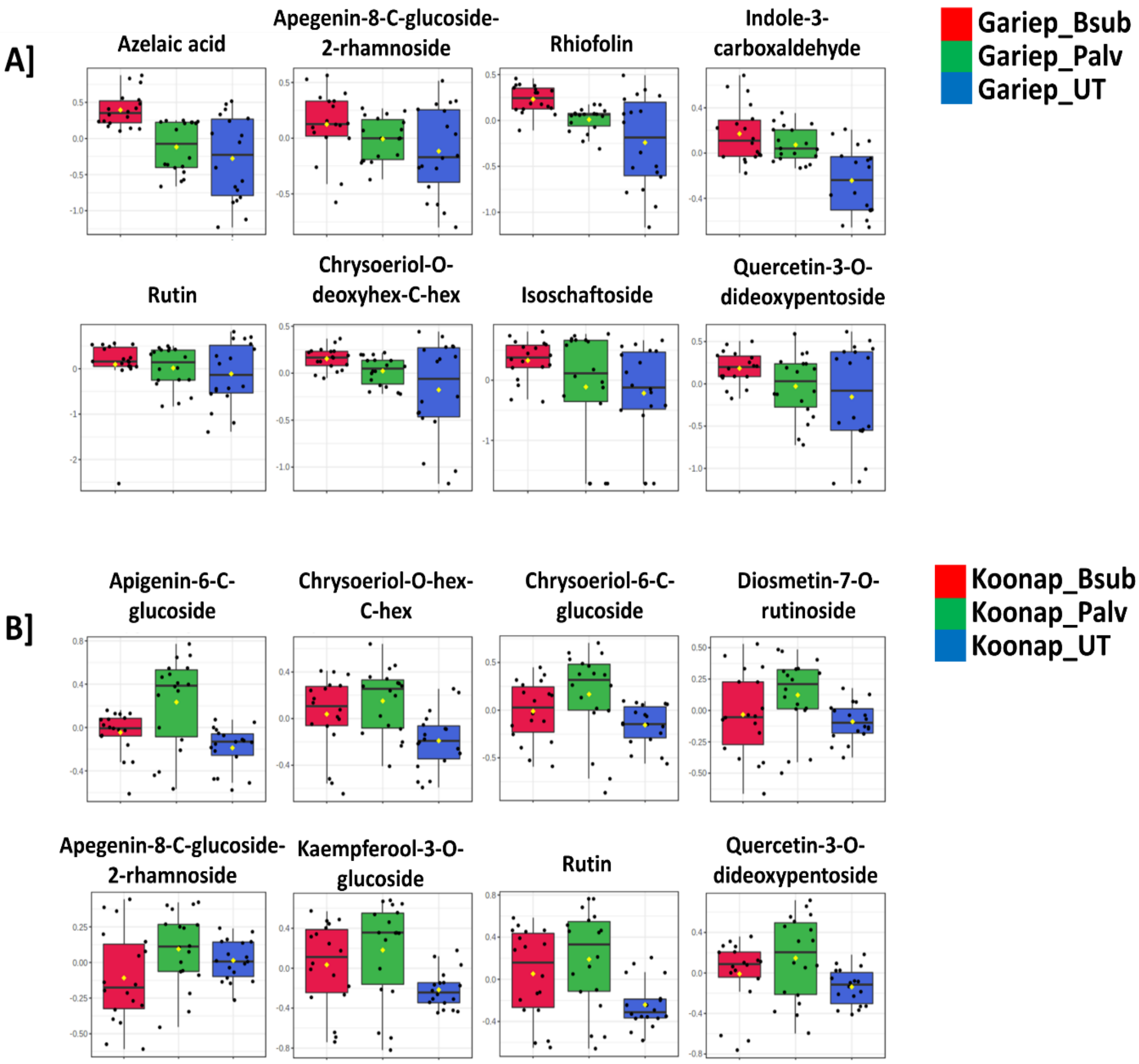

Figure S4: Differential quantitative profiles of increased annotated and metabolites. (**A**) and (**B**) show the differential accumulation of selected metabolites in the Gariep and Koonap cultivars respectively. The box and whiskers plots reveal an increase in phenolic compounds (phenylpropanoid), organic acids and benzenoids in the leaves of PGPR-treated Gariep (**A**) and Koonap (**B**) plants. The data were median-normalised, log transformed and *Pareto*-scaled. Red and green box and whiskers represent *B. sub* and *P. alvei* (T22) treatments, blue represents the untreated samples of wheat cultivars.

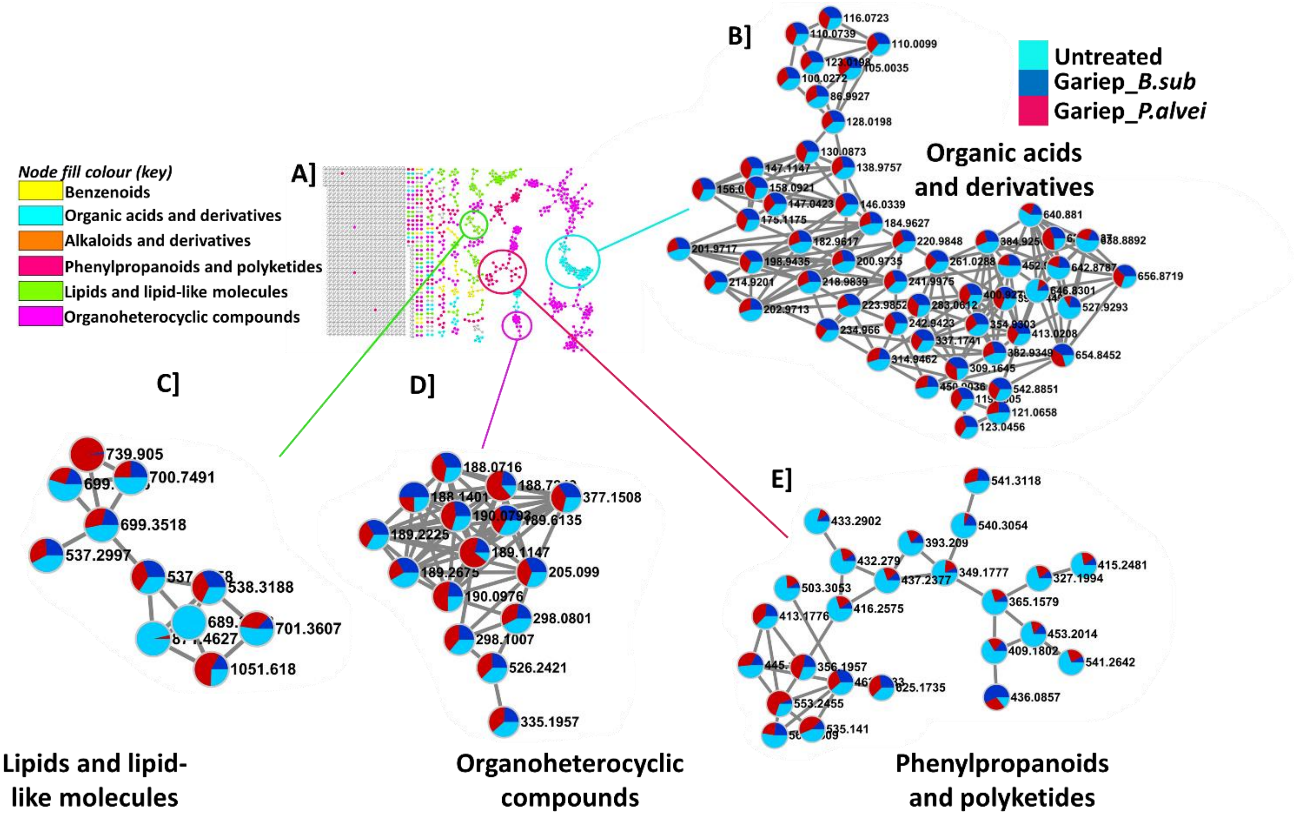

Figure S5: A global leaf metabolome view of the PGPR-treated/untreated Gariep rhizosphere showing classes and distribution of detected metabolites driving the global differences between the treated and untreated rhizosphere soils. (A) Molecular network of MS/MS spectra generated with MolNetEnhancer (in GNPS) giving a metabolome coverage and classes of extracted metabolites from rhizosphere soil of PGPR-treated and untreated Gariep cultivar. Each displayed node represents a metabolite, while each cluster of pooled nodes (coloured) depicts a class of chemically related and putatively annotated metabolites matched to GNPS libraries and databases. Grey nodes represent unmatched spectral data. **B, C, D** and **E** show clusters of organic acids and derivatives, lipids and lipid-like molecules, organoheterocyclic compounds and phenylpropanoids and polyketides respectively. Each node representing matched spectral data shows the differential distributions and changes in the metabolites per treatment.

Table S1: Summary of the annotated, putatively identified metabolites (MSI-L2) from Gariep and Koonap wheat cultivars.

No	Compound name	Chemical formula	Rt	*m/z*	Adduct	Fragments (*m/z*)
1	Indole-3-carboxaldehyde	C9H7NO	0.74	146.0304	[M+H]+	118
2	Oxaloacetate	C4H4O5	0.75	131.9753	[M-H]-	87
3	Rosmarinic acid	C18H16O8	0.80	163.0213	[M+H]+	145, 135, 117, 89
4	(p-Hydroxybenzyl) malonic acid	C10H10O5	0.81	209.949	[M-H]-	165, 163, 147
5	Adenine	C5H5N5	0.92	136.0707	[M+H]+	119
6	cis-Aconitate	C6H6O6	0.94	173.007	[M-H]-	173, 129, 85
7	Isocitric acid	C6H8O7	0.95	191.0186	[M-H]-	173, 155, 111
8	Citric acid	C6H8O7	0.96	191.0166	[M-H]-	173, 111
9	Fumaric acid	C4H4O4	0.98	115.0097	[M-H]-	71
10	Itaconic acid	C5H6O4	1.01	129.018	[M-H]-	129, 85
11	Malic acid	C4H6O5	1.04	133.0105	[M-H]-	115
12	L-Tyrosine	C9H11NO3	1.17	182.081	[M+H]+	165, 136, 123, 91, 56
13	Aconitic acid	C6H6O6	1.28	173.0081	[M-H]-	129, 117, 85
14	Succinic acid	C4H6O4	1.28	117.0192	[M-H]-	72
15	4-Fumarylacetoacetic acid	C8H8O6	1.81	199.006	[M-H]-	137, 135, 113, 101, 97, 83
16	p-Coumaraldehyde	C9H8O2	1.86	147.0437	[M-H]-	147, 93
17	Phenylalanine	C9H11NO2	1.86	166.085	[M+H]+	120
18	Indoline	C8H9N	1.92	120.08	[M+H]+	103
19	Benzoic acid + 2O, O-Hex	C13H16O9	1.97	315.0678	[M-H]-	153, 109
20	Phenylalanine	C9H11NO2	2.03	164.09	[M-H]-	147, 129, 103
21	Phenylalanine	C9H11NO2	2.03	164.09	[M-H]-	147, 129, 103
22	Alanylleucine	C9H18N2O3	2.54	203.139	[M+H]+	157, 132, 86
23	Tryptophan	C11H12N2O2	3.03	205.09	[M+H]+	188, 146, 118
24	Indole-3-carboxaldehyde	C9H7NO	3.03	146.06	[M+H]+	118
25	Monoethyl phthalate	C10H10O4	3.34	193.048	[M-H]-	149
26	Valine	C5H11NO2	3.35	118.0661	[M+H]+	70, 55
27	Tryptophan	C11H12N2O2	3.37	203.0699	[M-H]-	159, 142, 116
28	Glutamic acid	C5H9NO4	4.19	148.0577	[M+H]+	130, 84, 72
29	Leucine	C6H13NO2	4.27	132.1	[M+H]+	86, 69
30	Ascorbic acid	C6H8O6	4.81	177.0553	[M+H]+	163, 147, 145, 134, 127, 117, 89
31	4-(sec-Butoxy)benzoic acid	C11H14O3	5.06	217.0956	[M+H]+	144
32	Coumaroyl agmatine	C14H20N4O9	6.24	277.16	[M+H]+	260, 218, 147, 145, 131, 114
33	4-Hydroxymandelonitrile	C8H7NO2	7.12	150.0557	[M+H]+	122, 95
34	4-Acetyl-2(3H)-benzoxazolone	C9H7NO3	7.12	178.0495	[M+H]+	150, 122, 95
35	HMBOA + O-Hex	C15H19NO9	7.12	356.0928	[M-H]-	194, 138
36	HMBOA + O-Hex isomer	C15H19NO9	7.12	356.0951	[M-H]-	194, 166, 138, 122
37	4-Hydroxymandelonitrile	C8H7NO2	7.13	150.0564	[M+H]+	122
38	N-Acetylaspartylglutamate	C11H16N2O8	8.06	303.0881	[M-H]-	303, 96
39	Caffeyl alcohol	C9H10O3	9.12	165.053	[M-H]-	147, 119, 103

40	1-O-Sinapoyl-β-D-glucose	C17H22O10	9.81	385.1113	[M-H]-	223, 164
41	Quercetin-3-O-dideoxypentoside	C25H26O15	10.46	593.149	[M-H]-	447, 309 (-301), 285
42	Rutin	C27H30O16	10.54	609.1488	[M-H]-	593, 447, 309 (-301), 285
43	D-Tartaric acid	C4H6O6	10.84	149.01	[M-H]-	130, 89, 74, 59
44	Isoschaftoside	C26H28O14	10.86	563.1441	[M-H]-	473, 353; 325
45	Kaempferol-3-O-glucoside	C21H20O11	11.4	447	[M-H]-	285, 284, 255, 227
46	6,8-di-C-Glucosyl apigenin	C27H30O15	11.62	593.1588	[M-H]-	575, 473, 372
47	Naringenin	C15H12O5	11.72	271.063	[M-H]-	187, 177; 151, 119
48	Chrysoeriol-O-hexoside-C-hexoside	C28H32016	11.74	623.16	[M-H]-	443, 341
49	Apigenin 6-C-glucoside	C21H20O10	11.84	431.098	[M-H]-	413, 335, 311
50	Rhoifolin	C27H30O14	11.90	577.1522	[M-H]-	457, 413, 341, 269
51	Apigenin-8-C-glucoside-2'-rhamnoside	C27H30O14	11.92	579.164	[M+H]+	433, 313
52	Kaempferitrin	C27H30O14	12.13	577.1526	[M-H]-	563, 453, 431, 413, 355, 341, 293, 283
53	Chrysoeriol-O-deoxyhexoside-C-hexoside	C28H32O15	12.25	607.164	[M-H]-	461, 443, 425, 341, 311
54	Chrysoeriol 6-C-glucoside	C22H22O11	12.28	461.107	[M-H]-	443, 365, 341, 311
55	Diosmetin-7-O-rutinoside	C28H32O15	12.29	609.1782	[M+H]+	463, 301
56	Ribonic acid	C5H10O6	12.39	165.054	[M-H]-	149, 131, 119, 103, 73, 59
57	Azelaic acid	C9H16O4	13.41	187.093	[M-H]-	125, 97
58	Gallic acid monohydrate	C9H16O4	13.71	187.0958	[M-H]-	169, 125
59	Kaempferol	C15H10O7	13.88	285.078	[M-H]-	227, 151, 120
60	Salicylic acid beta-D-glucoside	C13H16O8	14.84	299.0932	[M-H]-	281, 255, 119, 93
61	(10E,15Z)-9,12,13-Trihydroxy-10,15-octadecadienoic acid (9,12,13, TriHODE)	C18H32O5	17.27	327.209	[M-H]-	229, 211, 183, 171, 113
62	Cinchonidine	C19H22N2O	19.70	293.1716	[M-H]-	236, 221
63	9-Hydroxy-12-oxo-10(E),15(Z)-octadecadienoic acid isomer II	C18H32O3	20.96	309.2026	[M-H]-	291, 247, 165
64	Thymol-beta-D-glucopyranoside	C16H24O6	22.29	311.1692	[M-H]-	183
65	Brefeldin A (Decumbin)	C16H24O4	22.33	279.162	[M-H]-	261, 225, 183, 197
66	Methyl (9Z)-10'-oxo-6,10'-diapo-6-carotenoate	C20H24O3	22.38	311.167	[M-H]-	279, 119, 79
67	Octadecenedioic acid	C18H32O4	22.4	311.166	[M-H]-	277
68	Valdiic acid	C17H26O5	22.42	309.173	[M-H]-	293, 225, 153, 97
69	Dirhamnosyl linolenic acid	C28H48O11	22.48	559.3103	[M-H]-	277 (-282)
70	Boldine	C19H21NO4	23.37	325.1827	[M-H]-	183
71	trans-Feruryltartaric acid	C14H14O9	23.46	325.183	[M-H]-	193, 119
72	Hydroxyoctadecatrienoic acid	C18H30O3	23.47	293.177	[M-H]-	277, 249, 152, 113, 97
73	Dehydrotumulosic acid	C31H48O4	24.03	484.721	[M+H]+	395
74	Oleamide	C18H35NO	25.34	282.2773	[M+H]+	265, 247, 184, 149, 95

75	Docosapentaenoic acid	C22H34O2	25.83	369.2971	[M+H]+	325, 124
76	2-Acetoxy-4-pentadecylbenzoic acid	C24H38O4	27.03	413.2596	[M+Na]+	301
